# Report from the 5th International Symposium on Mycotoxins and Toxigenic Moulds: Challenges and Perspectives (MYTOX) Held in Ghent, Belgium, May 2016

**DOI:** 10.3390/toxins8050146

**Published:** 2016-05-12

**Authors:** Sarah De Saeger, Kris Audenaert, Siska Croubels

**Affiliations:** 1 Department of Bioanalysis, Laboratory of Food Analysis, Faculty of Pharmaceutical Sciences, Ghent University, 9000 Ghent, Belgium; 2 Department of Applied Biosciences, Faculty of Bioscience Engineering, Ghent University, 9000 Ghent, Belgium; kris.audenaert@ugent.be; 3 Department of Pharmacology, Toxicology and Biochemistry, Laboratory of Pharmacology and Toxicology, Faculty of Veterinary Medicine, Ghent University, 9820 Merelbeke, Belgium; siska.croubels@ugent.be

## 1. Preface

The association research platform MYTOX “Mycotoxins and Toxigenic Moulds” held the 5th meeting of its International Symposium in Ghent, Belgium on 11 May 2016. The Symposium welcomed over 100 scientists, researchers and representatives from industry and government as well as academia to discuss all aspects of mycotoxin research including production, occurrence and detection, the impact on human and animal health, reduction and prevention, toxicology and other topics.

Mycotoxins—toxic fungal secondary metabolites—play a significant role in food and feed safety, as well as in medical and environmental microbiology. Indeed, mycotoxins have been shown to be the number one threat amongst food and feed contaminants regarding chronic toxicity. Economic losses are due to effects on livestock productivity and direct losses in crop yield and stored agricultural products. Legislative limits for a range of mycotoxins continue to develop worldwide, resulting in an increased number of official controls deriving from national food safety plans and for food trade purposes. Furthermore, environmental mycotoxins are a continuous threat to human and animal health.

The challenges presented to those working in mycotoxin research are enormous due to the frequency, the complexity and variability in occurrence. Several aspects make the pre- and post-harvest control of mycotoxins difficult, such as:

- Different fungal species produce mycotoxins;
- Most of the mycotoxin producing fungi are able to produce more than one mycotoxin;
- Mycotoxin levels are influenced by environmental conditions during growth and storage;
- The presence of modified mycotoxins;
- The highly complex influence of environmental factors on the biosynthesis of mycotoxins by fungi.

Other aspects related to human and animal health also contribute to the complexity in mycotoxin research, e.g.,

- The lack of suitable biomarkers to assess exposure of humans and animals;
- The need for guidance levels of mycotoxins in animal body fluids;
- The efficacy and safety testing of mycotoxin detoxifiers;
- Knowledge about toxicokinetics in men and animals.

New developments in mycotoxin analysis focus on faster, multi-mycotoxin, environmentally friendly, cost-effective and fit-for-purpose methods in food, feed, biological tissue and body fluids. A trend towards untargeted metabolic profiling has been noticed.

Mycotoxigenic fungi, mycotoxins and food and feed safety will continue to be a critical interest to researchers for years to come. Likewise, the risks for human and animal health by indoor air contamination and other sources of environmental mycotoxin exposition are still a wide field of research. Innovations take place at a rapid pace. The investigational area is broad (phytopathology, analytical methods, risk management, toxicology, biomarkers for exposure, occupational health risks), but necessary to ensure progress into improvement of public health and animal health, in particular a safe food and feed supply.

Only through multidisciplinary efforts and concerted actions can further progress and solutions be expected for the mycotoxin problem.

The association research platform MYTOX "Mycotoxins and Toxigenic Moulds" was established in 2007 and consists of more than 50 researchers from 12 research laboratories in the Ghent University Association. MYTOX deals with mycotoxin research in a multi-disciplinary way, based on four main units: (1) mycotoxins; (2) toxigenic fungi; (3) mycotoxins and animal health; and (4) mycotoxins and human health. In this way, MYTOX tackles the mycotoxin issue along the production chain from the field to the end consumer, within the ‘One Health’ concept.

The scientific program included oral and poster presentations related to fungal-related disease monitoring; mycotoxin analysis in food and feed, as well as in animal and human biological samples; prevention on the field; management strategies during food and feed storage and processing.

## 2. Keynote Lectures

### 2.1. Determination of Mycotoxins in Food and Feed—Quo Vadis?

Stroka, J.

Over the last decade, the number of mycotoxins that are regulated in Europe has steadily increased with the aim of protecting consumers as well as recognizing animal welfare. In parallel scientific progress in many fields such as bio-chemical analysis as well as technical-instrumental progress increased the possibilities to determine mycotoxins with completely new defined scopes, such as shorter time of analysis, parallel determination of mycotoxins, or improved detection capability (LOD/LOQ), while the most important aspect of every chemical determination remained the reliability of the generated value.

The European Union Reference Laboratory (EURL) for mycotoxins has, together with its partner from the national reference laboratories, not only monitored but also evaluated the performance of analytical methods with the aim ensure a reliable measurement capacity in Europe.

This presentation gives an overview of the most important achievements of the work of this network, demonstrated in data generated in the network and gives an outlook how the performance of analytical methods might need to be viewed taking the analytical progress into account and where routes for future improvements might be found.

### 2.2. Integrated Management of Mycotoxins in Pre- and Post-Harvest and MycoKey Solutions

Logrieco, A.F.

Among the emerging issues in food safety, the increase of plant diseases associated with the occurrence of mycotoxigenic fungal species is of major importance. As a result of their secondary metabolism, these fungi can produce mycotoxins, which are low-molecular-weight toxic compounds that represent a serious risk for human and animal health worldwide. The management of good agricultural practices in the pre-harvest is a key issue for minimizing the risk of mycotoxin accumulation in the crops before the harvest. Such practices can involve crop rotation, tillage, proper fertilization and fungicide or biological control distribution, variety selection, timely planting and harvests and the control of the insects which often act as vectors of toxigenic fungi spores. On the other hand, the reduction of mycotoxins along the agro-food chains is also highly dependent on a correct post-harvest management that must aim firstly at the separation of the infected crop products from the healthy material. Therefore, the use of different tools such as manual sorting or optical sensors is also a crucial point for reducing the level of mycotoxin contamination of a given crop. Moreover, it is extremely important to prevent post-harvest contamination during the storage by obtaining low temperature and humidity conditions, in order to limit the development of toxigenic fungal genera. An updated review of an integrated management of pre- and post-harvest practices aiming at minimizing the risk of mycotoxin contamination of the main crops of agro-food importance and main effective solutions proposed by EU project MycoKey will be provided in the presentation.

Acknowledgements: this presentation has been supported by the EU Project MycoKey N. 678781.

### 2.3. Analytical Management Strategies to Reduce Mycotoxin Contamination: A View from the Food Industry

Suman, M.

Mycotoxin contamination in various crops is of major concern since it has significant implications for food and feed safety, food security and international trade. Grain and foods based on grains (e.g., pasta, bread, bakery products) account for the largest contribution to mycotoxin exposure in all age classes. It has been estimated by the Food and Agricultural Organization (FAO) that, worldwide, approximately 25% of the crops are contaminated by molds: the incidence can vary considerably depending on many factors, such as weather conditions, agricultural practices, packaging, transport and storage. Results using the latest state-of-the-art multi-analyte methods show that almost 100% of these crops is contaminated with one or more mycotoxins.

Furthermore, in the next decades, industry will have to face a new “mycotoxins scenario”: the so-called “masked/bounded forms”. In fact, even if most mycotoxins demonstrate stability at room temperature and under neutral conditions, many factors (e.g., heat, pressure, pH, enzymatic activities, food constituents) must be considered during processing, since the release of native forms as well as formation of conjugated ones by reactions with macromolecular components (such as sugars, proteins or lipids) may be induced. Therefore, nowadays, the food industry clearly demonstrates the need for both rapid screening techniques, which could be also used outside the laboratory environment, and high sensitivity-precision methods for confirmatory purposes. Rapid and user-friendly Lateral Flow Devices (LFD) have been recently made available on the market: sensitivity and selectivity are provided by specific antibodies; generally, they do not require expensive instrumentation and additional chemicals or handling steps, providing in a few minutes a quantitative result for a target mycotoxin. LFD is in fact becoming progressively competitive with the common ELISA methods (Enzyme-Linked Immunosorbent Assay) which still combine good throughput performances for large numbers of samples and simple extraction-detection procedures. In the field of immunochemical methods, there is also a growing interest towards homogeneous approaches like the Fluorescence Polarization Immunoassay (FPIA) and optical-based detection provided by Surface Plasmon Resonance Sensors (SPRS).

Other emerging strategies are related for instance to near-infrared (NIR) spectroscopy and Electronic Noses. An Electronic Nose consists of an array of non-specific chemical detectors that interact with different volatile compounds and provide signals that can be utilised effectively as a fingerprint of the volatile molecules rising from the analysed samples.

Higher accuracy and repeatability can still be guaranteed by chromatographic based techniques like HPLC (High Performance Liquid Chromatography) with ultraviolet (UV) or fluorescence detection (FLD): pre-concentration and clean-up steps allow the achievement of good performances in very complex matrixes.

Liquid Chromatography coupled with Mass Spectrometry (LC-MS) is actually the most flexible and effective (high sensitivity and selectivity) technique used in order to determine mycotoxins in many different matrixes. At the same time, the natural coexistence of several mycotoxins imposes the need to optimize the global cost of these analyses through the development of multiresidual analytical strategies; in this contest, the new frontier is the High Resolution Mass Spectrometry (HRMS), in particular exploiting high performance Time of Flight (TOF) and Orbitrap^TM^ technology instruments, coupled to Ultra High Performance Liquid Chromatography (UHPLC): in this way, a door is opened to the simultaneous quantification of different categories of chemical contaminants.

### 2.4. Stepping out of the Dark; How Genomes and Genomics Shed Light on Biosynthetic Pathways

Van der Lee, T.; Hoogendoorn, K.; Medema, M.; Waalwijk, C.

New sequence technologies radically changed sequencing costs, sequencing quality and the time required for the completion of sequencing projects. Recently, high quality genome assemblies and gene annotations were generated for several *Fusarium* species (*F. graminearum*, *F. poae*, *F. culmorum*, *F. pseudograminearum*, *F. langsethiae* and *F. fujikuroi*). This provides unique opportunities to understand the function, organization and dynamics of the genes and chromosomes in these genomes. Our research focusses on genes that encode proteins involved in the production of fungal natural products such as mycotoxins. Although fungal natural products, sometimes referred to as ‘biosynthetic dark matter’, show an impressive variation in chemical structures and biological activities, their biosynthetic pathways share a number of key characteristics. First, genes encoding successive steps of a biosynthetic pathway tend to be located adjacently on the chromosome in biosynthetic gene clusters (BGCs). Second, these BGCs are often are located on specific regions of the genome and show a discontinuous distribution among evolutionarily related species and isolates. Third, the same enzyme (super)families are often involved in the production of widely different compounds. Fourth, genes that function in the same pathway are often co-regulated, and therefore co-expressed across various growth conditions. The availability of high quality genome assemblies where chromosomes are assembled from telomere to telomere, allow the precise localization and organization of genes that was often lacking from the previously available draft assemblies. The use of the millions of reads generated by RNA-seq data can result in improved annotation of genes that are lowly expressed as is typical for genes involved in the production of secondary metabolites analysis. Computational tools are indispensable to the discovery process, as they are required for the effective exploitation of genomic data to identify potential new secondary metabolites or variants of known natural products. They also can direct functional characterization of the biosynthetic routes. A key step in natural product bioinformatics is the identification of BGCs that encode the enzymatic pathways for compound production, as well as the prediction of their products. Frequently used computational tools for fungal BGC identification, such as antiSMASH, SMIPS/CASSIS and SMURF have largely focused on known types of BGCs, encoding the enzymatic pathways to produce compounds such as polyketides, nonribosomal peptides (NRPs) and terpenes. The intrinsic disadvantage of these programs is that they cannot detect types of BGCs that they were not trained to predict. Recently, algorithms have emerged that also allow the identification of additional, more obscure, BGCs. We will describe the identification and bioinformatic characterization of BGCs in these newly sequenced *Fusarium* genomes with the help of systematized information on BGCs that have been experimentally characterized in the past.

## 3. Presentations

### 3.1. Carry-Over of Aflatoxin B_1_ from Dairy Cows’ Feed to Milk

Van der Fels-Klerx, H.J.; Camenzuli, L.

Ingredients used in the cows’ diet, such as maize, can sometimes be heavily contaminated with aflatoxin B_1_. This mycotoxin was present in high amounts in maize grown in Italy in 2003 and 2008 and in maize grown in the Balkan area in 2013. Aflatoxin B_1_ is metabolized by dairy cows into aflatoxin M_1_ appearing in the cows’ milk. Aflatoxin B_1_ and aflatoxin M_1_ are carcinogenic to animals and humans, hence, within the European Community (EC) the presence of both these toxins is regulated. Nowadays, milk production of dairy cows is increasing, as is the rate of maize used in compound feed production for dairy cows. High producing cows were indicated to have a higher transfer rate of the mycotoxin and, consequently, this may affect the appearance of aflatoxin M_1_ in the cows’ milk.

This study aimed to estimate concentrations of aflatoxin M_1_ in dairy cows’ milk, given contamination of compound feed with aflatoxin B_1_ and feeding consumption regimes. Monte Carlo simulation modeling was applied, with 1000 iterations for each run. The model simulated two types of typical Dutch dairy herds composed of 69 cows. In the first type, all cows started their lactation at the same time, while in the other type, the start date of lactation was spread over the year, with up to two new cows starting lactation each week. The composition of a typical compound feed for dairy cows was used. National monitoring data were used to determine the concentration range of aflatoxin B_1_ in each of the ingredients. In addition to this ‘standard’ contamination, we also used the concentrations of the toxin in maize as reported from the Balkan incident in 2013. Six different equations from literature were used to assess the transfer of aflatoxin B_1_ in the cow’s body from feed to milk.

Modeling results showed that, in all scenarios considered, the maximum average aflatoxin M_1_ concentration in milk produced at the farm (all 69 cows) is below the EC legal limit of 0.05 µg/kg. In some scenarios, however, this maximum was close to this limit (up to 0.04 µg/kg). Apparently, the higher amount of milk produced also dilutes the aflatoxin M_1_ in the milk.

### 3.2. Biodegradability of Mycotoxins during Anaerobic Digestion

De Gelder, L.; Audenaert, K.; Willems, B.; Schelfhout, K.; De Saeger, S.; De Boevre, M.

When exceeding maximum limits or guidance values, highly-mycotoxin contaminated food and feed batches are excluded from the Flemish market. To date, it is mandatory by The Public Waste Agency of Flanders (OVAM), which dictates the fate of all waste streams and their appropriate removal, that these contaminated batches should be destroyed by combustion. In light of their policy in achieving maximum valorization of waste streams, OVAM wishes to explore the remediation of mycotoxin contaminated food and feed batches through anaerobic digestion.

Anaerobic digestion, mediated by a consortium of micro-organisms resulting in the production of biogas, is widely employed to treat organic-biological waste (OBW) and manure. In Flanders, 40 biogas plants are in operation or under construction with a total processing capacity of 2.234 million tons per year, of which 9 are thermophilic and 31 are mesophilic installations. OBW comprises 60% of the input in anaerobic digesters but still specific Flemish legislation concerning the presence of certain contaminants in the waste should be respected. Specifically, for mycotoxins, OVAM rejects the processing of OBW in anaerobic digesters due to insufficient guarantees regarding (i) the effect of the mycotoxins on the anaerobic digestion process itself; and (ii) the residual concentrations of mycotoxins which impairs the subsequent usability of the digestate, such as fertilizer or soil improver in agriculture.

In order to address these concerns, lab scale degradation tests were conducted to assess the biodegradability of aflatoxin B1, deoxynivalenol, zearalenone, fumonisin B1, ochratoxin A, T-2 toxin and ergot alkaloids during thermophilic and mesophilic digestion. In 20-day batch tests, where digestate was fed with fructose and spiked with each mycotoxin separately, more than 90% degradation was obtained for aflatoxin B1, deoxynivalenol, zearalenone and ochratoxin A. Remarkably, ergot alkaloids were only degraded for *ca.* 60% under mesophilic conditions but for more than 97% under thermophilic conditions. The presence of the mycotoxins did not result in a significant decrease in methane production. Subsequently, a naturally highly contaminated maize batch (4413 µg/kg deoxynivalenol, 1052 µg/kg zearalenone, 170 µg/kg fumonisin B1+B2) was fed to mesophilic and thermophilic semi-continuous reactors on pilot scale. After one hydraulic retention time (30 days), no mycotoxins were detected except for a small residue of 90 µg/kg deoxynivalenol in the thermophilic reactor. Also, reactor performance and methane production were not hampered by the prolonged feeding of the highly contaminated maize.

These results indicate that anaerobic digestion is generally a safe strategy for the remediation of mycotoxin contaminated food and feed batches.

### 3.3. Thermal Degradation of T-2 and HT-2 Toxins during Extrusion Cooking in Laboratory and Industrial Scale

Schmidt, H.S.; Becker, S.; Cramer, B.; Humpf, H.-U.

Trichothecene mycotoxins are naturally and worldwide occurring food contaminants, exposing humans and animals to adverse health effects. T-2 and HT-2 toxins are the most prominent members of Type A trichothecenes and are produced by several species of the *Fusarium* genus and are frequently found in oats, which is an increasing problem in times of a growing interest on oat-derived products for a healthy and balanced diet. Concentrations of the sum of both toxins in products for human consumption may exceed the EU-guidance levels of 200 µg/kg, which is regarded critically, due to the high toxic potential of both secondary metabolites.

There are several methods reported to reduce the mycotoxin content in food, ranging from agricultural strategies to prevent the fungal infestation to food processing steps that either remove the toxins or destroy the mycotoxins’ molecular structure. Food extrusion is a high-temperature short-time (HTST) process and was shown to efficiently lower the mycotoxin content due to a combination of high temperatures and pressure and severe shear forces.

We therefore investigated the stability of T-2 and HT-2 during food extrusion of toxin-spiked oat flour on a laboratory food extruder with samples differing in water content, die-temperature, pressure and shear rates applied by the screw. The results were compared to an extruder working on industrial scale with naturally contaminated oatmeal, also regarding the formation of degradation products.

### 3.4. Influence of Maturity Stages, Processing of Maize on Aflatoxins and Fumonisins Levels and Dietary Intake by Some Populations of Cameroon

Nguegwouo, E.; Njumbe Ediage, E.; Njobeh, P.B.; Medoua, G.N.; Ngoko, Z.; Fotso, M.; De Saeger, S.; Fokou, E.; Etoa, F.-X.

Food safety is a call for concern nowadays with mycotoxin contamination particulary aflatoxins and fumonisins as a contributing factor. The aim of this research was to investigate the effect of maturity stage of maize at harvest, post-harvest handling and processing techniques on total aflatoxins (AFT): AFB_1_, AFB_1_, AFG_1_, AFG_1_ and total fumonisins (FT): FB_1_, FB_2_, AFB_3_ as well as the dietary intake by some rural Cameroonian populations. Three stages of maturity as currently practiced (80, 85 and 90 days after seed), two drying processes after harvest (sun or barns drying up to two weeks), three periods of storage duration after drying (one, two and three months) and 11 types of maize products were considered during sampling. The analyses of AFT and FT were carried out using quantitative ELISA and those samples with level of mycotoxins higher than regulated level in the European Commission were analyzed by LC-MS/MS to know the sub-types of toxins present. To assess the dietary intake of those mycotoxins, a food consumption survey was conducted on 366 individuals that consisted of 108 children (4–8 years), 102 adolescents (9–14 years) and 156 adults (15 years and over). The results have shown that all analyzed samples were contaminated with AFT (range: 0.8 to 20.0 µg/kg) and FT (range: 100 to 5990 µg/kg). Sun or barn drying for only one week followed by one month usual storage resulted in significant FT contamination, emphasizing the need of at least two weeks of drying. The processing techniques reduced the levels of FT up to 94.74%, mainly in maize dishes that have a screening phase. The estimated intake of AFT and FT respectively showed that children are most at risk (mean: 43.8 × 10^−3^ and 13.2 µg/kg bw/day) followed by adolescents (mean: 31.9 × 10^−3^ and 9.0 µg/kg bw/day), and finally, adults (mean: 27.4 × 10^−3^ and 4.0 µg/kg bw/day).

### 3.5. Multi-Mycotoxins Occurrence in Maize and Animal Feed from Assiut Governorate, Egypt

Abdallah, M.F.; Kilicarslan, B.; Girgin, G.; Baydar, T.

Mycotoxins are secondary metabolites produced by different species of fungi such as *Aspergillus*, *Penicillium* and *Fusarium* with a broad range of toxic effects on human and animals. Over the last decade, several analytical methods have been developed for simultaneous detection of different mycotoxins in maize and feed in one run. Surveys of different countries around the world have shown the existence of the aflatoxins, ochratoxin A, and zearalenone in maize and animal feed. Relatively less information is available about the co-occurrence of different mycotoxins in Upper Egypt. Moreover, the current regulations in Egypt include only total aflatoxins and aflatoxin B1, 20 μg/kg and 10 µg/kg respectively, for both maize and animal feed. We therefore performed a survey of six mycotoxins, aflatoxins B1, B2, G1, and G2, ochratoxin A and zearalenone, in maize (*n* = 61) and animal feed (*n* = 17). Multi-mycotoxins immunoaffinity columns were used for clean-up and HPLC-FLD with on-line post-column photochemical derivatization was used for quantification of the mentioned mycotoxins.

A solid–liquid extraction has been done before passing the samples through the immunoaffinity columns. The chromatographic separation is based on the publication of Ofitserova *et al.* (2009). Limits of detection (LOD) were 0.92 µg/kg for ZEA, 0.02 µg/kg for OTA and varied from 0.004 to 0.12 µg/kg for aflatoxins. Limit of quantification (LOQ) were 2.8 µg/kg for ZEA, 0.06 µg/kg for OTA and varied from 0.013 to 0.3 µg/kg for aflatoxins. The mean recovery values were 77%–110% for different concentrations of AFs, OTA and ZEA in spiked maize and feed samples.

OTA, AFG1, and AFG2 were under the limit of detections. AFB1 was detected in both maize (*n* = 15) and feed (*n* = 8) with only one maize sample above the maximum permissible level set by Egyptian authorities. AFB2 was detected in six maize samples and in one feed with a maximum value of 0.05 µg/kg. ZEA was detected only in feed samples (*n* = 4) with a maximum value of 3.5 µg/kg. In conclusion, further surveys are highly recommended in order to establish database for mycotoxins occurrence in Egypt to minimize the possible health risks in animals and human.

Acknowledgements: this work is financially supported by Hacettepe University Scientific Research Projects Coordination Unit (#014 D06 301 002-620).

Ofitserova, M.; Nerkar, S.; Pickering, M.; Torma, L.; Thiex, N. Multiresidue mycotoxin analysis in corn grain by column high-performance liquid chromatography with post column photochemical and chemical derivatization: Single-laboratory validation. *J. AOAC Int*. **2009**, *92*, 15–25.

### 3.6. Effects of Climate Change on the Presence of Aflatoxin Producing Aspergillus Species in Central Europe

Baranyi, N.; Kocsubé, S.; Szekeres, A.; Bencsik, O.; Vágvölgyi, C.; Varga, J.

Global warming can affect the presence of thermotolerant fungi with potential mycotoxin producing abilities in our foods and feeds. In the past few years, this phenomenon was observed in several European countries which did not face this problem before. Furthermore, consequent aflatoxin contamination was observed in agricultural commodities including maize and milk in these countries. Aflatoxins are among the economically most important mycotoxins, they can cause serious yield loss in several agricultural products including cereals, cotton, maize and peanut. Aflatoxins are produced by various *Aspergillus* species mainly belonging to *Aspergillus* section *Flavi*. The economically most important producers of aflatoxins are *A. flavus*, *A. parasiticus* and *A. nomius*. While *A. flavus* produces B-type aflatoxins, *A. nomius* and *A. parasiticus* are also able to produce G-type aflatoxins. Other species able to produce G-type aflatoxins include, e.g., *A. bombycis*, *A. pseudonomius*, *A. parvisclerotigenus*, *A. minisclerotigenes* and *A. pseudocaelatus*. Although aflatoxin producers prefer tropical and subtropical climates, these species can occur in temperate climate in increasing quantities. These observations led us to examine the occurrence of mycotoxin producing Aspergilli in agricultural products in Hungary and its neighboring countries. Several potential aflatoxin producing isolates were found, *A. flavus* was the dominant. In this study we examined the aflatoxin producing abilities of *A. flavus*, *A. nomius*, *A. parasiticus* and *A. pseudonomius* isolates that came from cheese (Hungary), indoor air (Croatia), maize (Serbia, Hungary), and wheat (Hungary), respectively. We have found some differences in the ratio and amount of produced B- and G-type aflatoxins under different cultivation conditions in case of *A. nomius*, *A. pseudonomius* and *A. parasiticus* isolates.

Acknowledgements: present work was supported by OTKA grants No. K115690, K84122 and K84077. N.B was also supported by EMET grant No. NTP-EFÖ-P-15-0486 providing infrastructure.

### 3.7. Mycotoxin Exposure through Biomarker Analysis Using LC-MS/MS

Huybrechts, B.; Heyndrickx, E.; Sioen, I.; De Saeger, S.; Callebaut, A.

The aim of the presented study was to assess human mycotoxin exposure based on the direct measurement of urinary biomarkers via LC-MS/MS in samples of the Belgian population. Urine as target matrix has the advantage of being non-invasive while LC-MS opens the possibility of multi-analyte measurements. These biomarkers are often present in very low concentrations, so many methods published up till now rely on some sample treatment. Morning urine of 155 children (3–12 years) and 239 adults (19–65 years) was collected according to a standardised study protocol. These urine samples were analysed for the presence of 33 urinary mycotoxins and their metabolites. Nine out of 33 potential biomarkers were detected whereby deoxynivalenol (DON), DON-glucuronides (DON-GlcAs), deepoxy-deoxynivalenol-glucuronide (DOMGlcA), ochratoxin A, citrinin and dihydrocitrinone were the most frequently detected. DON15GlcA was the main urinary metabolite found in 100% of the samples and for the first time DOMGlcA was detected in urine of children. Screening a small number of samples via a more sensitive method using immuno-affinity column (IAC) based clean-up revealed the presence of AfM1 at pg·mL^−1^ levels in every urine. Fumonisins were not detected in the Belgian samples.

### 3.8. Effect of Thymol, 4-Hydroxybenzaldehyde, and Fluconazole on the Aflatoxin B1 Biosynthesis

Dzhavakhiya, V.G.; Voinova, T.M.; Statsyuk, N.V.; Shcherbakova, L.A.

In the course of evolution, plants affected by toxigenic fungi could develop some specific compounds to block the biosynthesis of fungal toxins. This supposition is confirmed by the phenomenon of the aflatoxin B1 (AFB1) biosynthesis inhibition by some terpenoids of plant origin. This group of biologically active substances includes thymol, which antimicrobial properties are widely used in human and veterinary medicine and plant growing. However, thymol has never been considered as a possible inhibitor of the AFB1 biosynthesis.

Our earlier studies showed that thymol and 4-hydroxybenzaldehyde (4-HBA), another compound of plant origin, represent highly efficient sensitizers of plant pathogenic fungi to fungicides. The revealed chemosensitizing effect is similar to the action of antioxidants and is connected with the inhibition of oxidative stress that increases the sensitivity of plant pathogenic fungi to some fungicides. In this study we investigated the ability of thymol and 4-HBA to inhibit AFB1 biosynthesis in *Aspergillus flavus*. In addition, the effect of fluconazole, a synthetic fungicide of a triazole group able to block melaninogenesis in *A. flavus*, on the AFB1 biosynthesis was studied; according to our hypothesis, since both melanin and AFB1 are synthesized by the polyketide pathway, this fungicide was probably able to block the toxigenesis.

According to the obtained data, the thymol addition to a liquid nutrient medium at concentrations of 0.05 and 0.075 µg/mL resulted in a significant reduction of the AFB1 production (79% and 71%, respectively) with the simultaneous blocking of melaninogenesis in fungal mycelium. The addition of 4-НВА (0.5 and 0.75 µg/mL) also inhibited the AFB1 production (90% and 92%, respectively) and caused the discoloration of fungal colonies. Unlike these two compounds, the addition of fluconazole at concentrations of 0.1 and 0.125 µg/mL increased the level of the AFB1 production by 93% and 179%, respectively, and, at the same time, blocked melaninogenesis.

It is known that AFB1 and melanin biosynthetic pathways have common initial stages and then diverge. Based on the obtained results, we can conclude that thymol and 4-HBA block those stages of the polyketide pathway, which are located prior the branch point, whereas fluconazole blocks the melanin biosynthesis stage located after the branch point that probably causes an increased accumulation of AFB1 precursors and the corresponding increase in the AFB1 production level.

This study was financially supported by the Russian Science Foundation (project No. 14-16-00150).

### 3.9. Microbial Degradation of Deoxynivalenol

Vanhoutte, I.; Audenaert, K.; De Saeger, S.; De Gelder, L.

Mycotoxin contamination of food and feed poses major risks for human or animal health and leads to economic losses. Prevention and intervention measures are very well described on the field, but still contaminated batches remain a reality in practice. In order to salvage these resources, feed remediation based on mycotoxin adsorption is already applied through the use of binders. However, adsorption is reversible, pH-dependent and non-specific. Moreover, these binders negatively influence the transfer of medication to the bloodstream. Therefore, there is need to develop more reliable detoxification strategies. This research focuses on the microbial degradation of mycotoxins, in particularly deoxynivalenol (DON) which frequently occurs in crops in Belgium. Several microbial communities, with a possible exposure history to mycotoxins or other complex molecules, are screened for the presence of DON degrading microorganisms. Enrichment cultures of soil and activated sludge showed degradation of DON after two weeks, as analyzed with ELISA, whereas detoxification of DON was confirmed with a bio-assay using *Lemna minor*. Subsequently, several strains, among which one promising isolate was related to *Streptomyces* sp. (derived from soil), have been purified and characterization of their degrading capacities is ongoing.

### 3.10. Quantitative UHPLC-MS/MS Method for Citrinin and Ochratoxin A: Prevalence in Food, Feed and Red Yeast Rice Food Supplements

Kiebooms, J.A.L.; Huybrechts, B.; Thiry, C.; Tangni, E.K.; Callebaut, A.

Mycotoxins have been reported to cause deleterious effects in animals and humans (a.o.: nephrogenic, hepatogenic, carcinogenic, teratogenic, and neurogenic). Some mycotoxins (e.g., aflatoxins and ochratoxin A) have already been well studied, but for citrinin, a nephrotoxic mycotoxin, this has not yet been the case. According to a recent European Food Safety Agency report, occurrence data are lacking for a correct risk assessment of citrinin. Besides, Belgian and German scientific reports have shown that citrinin or its metabolite, dihydrocitrinone are widely (in up to 90% of samples) present in human urine, which might imply chronic exposure. Recently, a maximum limit for citrinin was set in food supplements comprising red yeast (*Monascus purpureus*) fermented rice (RYR), which contains monacolin K, an active component against cholesterolemia. During fermentation the fungus can also produce citrinin. Consequently, the present work aimed to develop a robust and routinely applicable ultrahigh performance liquid chromatography-tandem mass spectrometry (UHPLC-MS/MS) method for the analysis of two incidentally co-occurring nephrotoxic mycotoxins, citrinin and ochratoxin A, in food, feed and in RYR food supplements. The method was successfully validated in RYR food supplements and wheat flour, achieving respective limits of quantification for citrinin of 0.6 µg/kg and 0.6 µg/kg and for ochratoxin A of 10 µg/kg and 0.6 µg/kg. The recoveries varied between 75% and 102%. Furthermore, a preliminary occurrence study in 135 different RYR, food and feed products was executed, proving the potential of this method for future data acquisition within a risk assessment framework regarding citrinin and ochratoxin A (co-)occurrence in food and feed matrices.

### 3.11. Degradation and Epimerization of Wheat Ergot Alkaloids during French Baking Test

Meleard, B.

With increasing reports of ergot sclerotia detection on cereal grains in EU, regulation (EC) No 1881/2006 has recently been amended to set a maximum level of ergot sclerotia in unprocessed cereals at 0.5 g/kg for human consumption. The wheat ergot sclerotia produce six major alkaloids with variable toxicological effects: ergotamine, ergosine, ergocristine, ergometrine, ergocornine, and ergocryptine. Due to epimerization each (*R*)-configured ergot alkaloid is associated to a (*S*)-configured epimeric form. The two epimers could differ regarding their toxicity with high toxicity of the (*R*)- form whereas the (*S*)- form could be biologically inactive. For this reason, the sum of 12 ergot alkaloids must be considered and the analysis of each specific epimer must be performed.

For our study, a sclerotia grinding containing 3.0 mg/g alkaloids was added to a commercial wheat flour (“*Corde Noire*”). Seven mixes were produced to obtain contaminated flour in a range of concentration for alkaloid from 60 µg/kg to 15,000 µg/kg.

A French baking test was performed at ARVALIS laboratory according to the standard method NF V03-716. The hydration rate is about 60% and cooking temperature is 250 °C. Alkaloid content was determined by means of LC-MS/MS at UGent Laboratory of Food Analysis in dough and bread after cooking and separately in crumb and crust.

For all flours the baking process led to a decrease of alkaloid content and to a conversion from the toxicologically relevant (*R*)-epimer to the biologically inactive (*S*)-epimer.

The degradation rate is variable according to the ergot alkaloid. In this experimentation ergotamine and ergosine were more lowered whereas ergometrine was more stable. The epimerization rate differs for each ergot alkaloid too; ergometrine and ergocornine were more sensitive to this phenomenon in our study. The epimerization increased with the temperature as shown by the decrease of the (*R*)- to (*S*)-epimer ratio from dough to crust.

As a conclusion, flour could be detoxified by both epimerization and degradation of ergot alkaloids. The alkaloid content in bread was reduced by 60% from the flour content whatever the alkaloid concentration was.

### 3.12. Identification and Classification of Agronomic Factors Involved in Ergot Contents and Its Alkaloids in Small Grain Cereals

Orlando, B.; Maumené, C.; Valade, R.; Maunas, L.; Robin, N.; Bonin, L.

The European Commission Regulation (EU) 2015/1940 of 28 October 2015 amending Regulation (EC) No 1881/2006 as regards maximum levels of sclerotia of ergot in certain unprocessed cereals has established maximum limits for ergot contents (*Claviceps purpurea*) in small grain cereals for human consumption.

Occurrence studies have shown that all the small grains cereals are affected by this disease. Triticale and rye are the most sensitive. Since 2012, ARVALIS—Institut du Végétal has studied more than 2000 farm fields of soft wheat, durum wheat, barley, rye and triticale according to the same methodology, in collaboration with partners.

This work allowed, on the one hand, the determination of the relationship between crop contamination with ergot and the associated production of alkaloids. The total alkaloid content is, on average, 0.32% with variation in μg of alkaloid/g of ergot from 57 to 36,385; that means from 0.006% to 3.6% of ergot bodies weight. The ratio between -ine and -inine forms is 2.6:1. Ergotamine, ergocristine, ergosine and their corresponding epimers represent 74% of total alkaloids. On average the content of total alkaloids of *C. purpurea* is 3103 μg/g. The host plant and the harvest year did not have influence on the alkaloid contents. The ergot contents explain 79% of the variability of alkaloid contents.

On the other hand, the survey allowed the identification and prioritization of some agronomic levers to limit the risk at crop level. A statistical analysis was applied on ln(total alkaloid content). The variance analysis applied to agronomic factors showed that the host plant, the previous crop, grassweeds presence and tillage system, significantly influence alkaloids contents. The relevance of these levers was confirmed by separate analytical works.

This work is a multifactor field prevention tool capable to limit and to manage the ergot and alkaloids risk. Nevertheless, we know that the main factor involved in ergot infection is the weather conditions.

### 3.13. Development of a Quantitative PCR Method Specific to Claviceps Purpurea, Ergot of Cereals: Applications to Study Its Dispersion and Evaluation of Its Toxicity

Dauthieux, F.; Vitry, C.; Ducerf, R.; Leclere, A.; Maumené, C.; Orlando, B.; Valade, R.

The resurgence of ergot of cereals in France is an important health issue due to the production of alkaloids in the sclerotia of *Claviceps purpurea.* A method for quantifying G1 group of *C. purpurea* (pathogenic subspecies of cereals and grasses) by real-time PCR was developed to provide an innovative and powerful tool in order to manage the risk associated with the ergot. The validated method was used to study dispersion of primary and secondary inoculum and to characterize flours for their ergot concentration in relation with their alkaloid content.

Insects were identified as potential vectors of ergot and we confirmed the low dispersion capacity of primary inoculum by wind. The advantage of the qPCR was also demonstrated in order to study the occurrence of the pathogen in flours and to link fungal biomass with the alkaloid content.

### 3.14. Effect of Fungicide seed Treatments on Germination of Claviceps Purpurea Sclerotia

Maunas, L.; Robin, N.; Maumené, C.; Janson, J.P.

Ergot of cereals (*Claviceps purpurea*) may be introduced into healthy parcel through seeds containing ergot sclerotia. These sclerotia are able to germinate in spring and release ascospores in the air. To reduce this source of pollution, a fungicide seed treatment could be a useful complementary tool to seed cleaning, by reducing the viability of the remaining sclerotia. Previous tests in controlled conditions identified potentially effective treatments. This study aims to measure the efficacy of these treatments in the field conditions. The results confirm the interest of two seed treatments, one containing prochloraz and triticonazole, and the other containing carboxin and thiram. They allow a high reduction of the stroma production and, potentially, of the spore production. Their efficacy is not ideal but it permits the possibility of a better control of the disease dispersion due to contaminated seeds.

### 3.15. Tillage, an Efficient Lever to Limit Ergot in Cereals

Maumené, C.; Orlando, B.; Labreuche, J.; Leclere, A.; Maunas, L.

Ergot of cereals (*Claviceps purpurea*) is conserved in the soil as sclerotia. After vernalization, these conservation bodies are able to germinate in the spring and to produce stroma, sorts of head with perithecia supported by a stalk. Buried deep these sclerotia are able to germinate, but fail to emerge and to release ascospores in the air. This work attempts to describe the depth distribution of sclerotia, artificially dispersed on the soil surface, under the influence of different tillage systems (plowing, shallow cultivation, combination of both) over a two-year sequence. It follows, depending on the depth of burial, an estimate of the risks and benefits associated with each single practice or combination. Following results are discussed and shown in Table 1.

After two tillages and almost 10 months later, 14% of the sclerotia were found. The double plowing leads to a relatively homogeneous distribution of sclerotia in the layers observed. It brings back to the surface (0–10 cm), up to 60% of recovered sclerotia. The realization of a single ploughing led to concentrate sclerotia below 10 cm depth. The efficacy (burying more than 10 cm) after two years is respectively to the sequences, L/WS and WS/L, of 81% and 64%. In both cases, less than 10% of sclerotia have been found in the layer 0–5 cm.

### 3.16. Modulation of Ochratoxin A Genes’ Expression in Aspergillus Carbonarius by the Use of Some Essential Oils

El Khoury, R.; Atoui, A.; el Khoury, A.; Cverheec, C.; Maroun, R.; Mathieu, F.

Ochratoxin A is a mycotoxin, mainly produced on grapes by *Aspergillus carbonarius*, that causes massive health problems for humans; therefore, its presence in food and feed is highly controlled. This study aims to reduce the occurrence of this mycotoxin using the 10 following essential oils (E.O): fennel, cardamom, anise, chamomile, celery, cinnamon, thyme, taramira, oregano and rosemary, by evaluating, on Synthetic Grape Medium (SGM), for 4 days, their effects on the growth of *A. carbonarius* S402 cultures and on its ability to produce OTA. Results showed that *A. carbonarius’* growth was reduced up to 100%, when cultured with the E.Os of cinnamon, taramira, oregano and thyme. As for the other six essential oils, their effect on *A. carbonarius’* growth was insignificant, but highly important on the OTA production. In fact, the fennel’s oil at 5000 ppm reduced the OTA production up to 88.9% compared to the control, with only 13.8% of growth reduction. These results led us to further investigate the effect of these E.Os on the expression levels of the genes responsible for the OTA production: *acpks*, *acOTApks*, *acOTAnrps* and the two regulation genes *laeA* and *vea*, using the qRT-PCR method. The results revealed that these six E.Os reduced the expression of the five studied genes: the *ackps* was the most downregulated, reaching 99.2% of inhibition with 5000 ppm of fennel’s E.O, while the other genes’ inhibition levels ranged between 10% and 96% depending on the nature of the essential oil and its concentration in the medium.

### 3.17. Fast and Sensitive Total Aflatoxins ELISA Development, Validation and Application for Food and Feed Analysis

Oplatowska-Stachowiak, M.; Sajic, N.; Xu, Y.; Mooney, M.H.; Gong, Y.Y.; Verheijen, R.; Elliott, C.T.

Aflatoxins (aflatoxin B_1_, B_2_, G_1_ and G_2_) produced by toxigenic fungi can contaminate different agricultural commodities such as corn and peanuts. Due to their toxic effects in humans and animals fast and validated methods for the detection of aflatoxins in food and feed are required for the identification of the contaminated batches before they are processed into final products and placed on the market. In order to address the need for improved detection methods seven monoclonal antibodies for aflatoxins with a good compromise between sensitivity and cross-reactivity were produced. Antibody showing IC_50_ of 0.031 ng/mL for AFB_1_ was applied in simple and fast direct competitive ELISA test for the detection of total aflatoxins. The developed ELISA kit was validated for peanut matrix. The detection capability was 0.3 µg/kg for aflatoxin B_1_, which is one of the lowest reported values. Critical assessment of the performance of the total aflatoxins ELISA kit for the detection of aflatoxins B_2_, G_1_, and G_2_ was also performed. The kit was used to analyze 32 peanut and peanut butter samples purchased locally and two samples containing small but detectable amounts of aflatoxin B_1_ were identified, which was confirmed by LC-MS/MS analysis. The test was further used to analyze 25 feed ingredients samples and 20 maize samples and the results were correlated with these obtained by LC-MS/MS method. The total aflatoxins ELISA kit was also tested in three proficiency schemes and it was demonstrated to have high accuracy. The developed assay has been transformed into commercial product for fast and easy detection of aflatoxins in food and feed.

### 3.18. Human Biomonitoring and Its Application to Mycotoxin Exposure Assessment: Revealing the Toxicokinetics of Deoxynivalenol in Humans

Heyndrickx, E.; Mengelers, M.; De Saeger, S.

Recently, a study by Heyndrickx *et al.* (2015) demonstrated the presence of several mycotoxins in urine samples (*n* = 394) of the Belgian population. Deoxynivalenol (DON) and especially its glucuronides were most prevalent, being present in 100% of the urine samples. A risk assessment was performed by deriving estimated dietary intakes from the urinary concentrations. The estimated intake for DON varied between 0.11–19.57 and 0.03–10.08 µg/kg bw/day for children and adults respectively. This could imply a health risk as 56%–69% of children and 16%–29% of the adults were estimated to exceed the tolerable daily intake for DON (1 µg/kg bw/day) depending on the approach applied. However, it has to be highlighted that there are still a lot of uncertainties when estimating the DON intake using urinary biomarkers due to the lack of toxicokinetic data of DON in humans.

For this reason, an intervention study with 14 adults was designed in order to obtain tentative information about the toxicokinetics of DON in humans. The design was partly based on a theoretical kinetic model developed for DON in humans. Prior to the start of this intervention study, each volunteer had to follow a DON restricted diet for 2 days. Additionally, each volunteer received a bolus of DON through a naturally contaminated breakfast while remaining on the DON restricted diet for the rest of the day. Urine samples were collected at different time points in the following 24 h. The urine samples were analysed for the presence of DON as well as its major metabolites de-epoxy-deoxynivalenol, deoxynivalenol-3-glucuronide and -15-glucuronide, using a targeted LC-MS/MS method. As the presence of unknown metabolites can lead to an underestimation of the exposure, additionally an untargeted screening was performed using HR-MS.

Toxicokinetic parameters such as the excretion pattern of DON and metabolites throughout a day and absorption and elimination rates will be calculated using the concentration-time curves in urine. Furthermore, this study could give a decisive answer about the use of morning or spot urine compared to 24-h urine and for the first time natural inter-individual variations will be determined within this group of volunteers. The obtained knowledge will serve to develop a standardised method to estimate DON intake by means of biomarker analysis.

Acknowledgements: this research was financially supported by the Research Foundation Flanders (grant number G.0D4615.N) as part of ‘The Food Biomarkers Alliance project (FOODBALL)’ within the ‘EU Joint Programming Initiative: A Healthy Diet for a Healthy Life: Biomarkers for Nutrition and Health’.

Heyndrickx, E.; Sioen, I.; Huybrechts, B.; Callebaut, A.; De Henauw, S.; De Saeger, S. Human biomonitoring of multiple mycotoxins in the Belgian population: Results of the BIOMYCO study. *Environ. Inter.* **2015**, *84*, 82–89.

### 3.19. Determination of Blood and Immune Parameters in Broilers Exposed to Fumonisin

Koppenol, A.; Junior, P.M.; Dhaouadi, S.; Caron, L.F.

Fumonisins (FUM) are mycotoxins commonly produced by molds belonging to the *Fusarium* genus. They are usually identified in poultry feed, because these feeds are mainly based on corn. One of the strategies currently available to mitigate the effect of mycotoxins in livestock is the inclusion of anti-mycotoxin additives, with specific adsorbent and/or detoxifying properties against those compounds.

The aim of the present trial was to assess the effects of feeding broiler chickens with FUM naturally contaminated feed and its impact on the poultry’s immune response and blood variables as well as to evaluate the protective effect of adding an anti-mycotoxin additive to this feed. In total, 96 male Cobb 500 day-old-chicks were divided over three isolators of 32 chicks each. Animals were fed one out of the three dietary treatments *ad libitum* until 28 days of age; A corn-soybean meal based control diet, a FUM contaminated diet (17 ppm) or a FUM contaminated diet supplemented with an anti-mycotoxin additive. The contaminated diet was formulated by replacing control corn by a naturally contaminated corn with *Fusarium* mycotoxins. The anti-mycotoxin additive supplemented diet was prepared using 0.2% of a commercial product (Elitox^®^).

Blood samples were taken from eight animals per treatment at 3, 7 and 14 days of age for evaluating circulating lymphocytes, as well as at 14 and 28 days of age for the determination of plasma proteins, albumin and globulin and serum levels of sphingosine (SO) and sphinganine (SA). On day 7, 14 and 28, samples of jejunum were collected from six animals per treatment for intestinal mucosa cell measurement.

Results showed that FUM had detrimental effects in broilers, resulting in decreased hematocrit and increased albumin, albumin/globulin and SA:SO values in the blood as well as decreased T-helper lymphocyte, activated T-cytotoxic lymphocytes and monocytes concentrations. The addition of the anti-mycotoxin additive was shown to ameliorate most of the negative effects of FUM.

### 3.20. Development of an LC-MS/MS Method for Simultaneous Determination of Beauvericin, Enniatins (A, A1, B, B1) and Cereulide in Cereal and Cereal-Based Food Matrices

Decleer, M.; Rajkovic, A.; Sas, B.; Madder, A.; De Saeger, S.

Beauvericin and the related enniatins are mycotoxins, secondary metabolites mainly produced by different *Fusarium* species that occur naturally on cereal and cereal-based foods and feeds. The emetic toxin cereulide is produced by the specific strains of *Bacillus cereus*. Structurally, these toxins are cyclic depsipeptides with ionophoric properties. Their toxicity is mediated by the ability to initiate cation transport across the cell membrane, disrupting normal intracellular cation levels, leading to apoptosis which is accompanied by DNA fragmentation. They are highly resistant to heat, acidification and proteolytic enzymes. Although these emerging foodborne toxins have different microbial origin, the striking structural and functional similarities enable and provide rationale for their concurrent detection in food matrices. Due to their prevalence in food and feed, and their toxicity it became an imperative to create a new tool in microbial food diagnostics for their reliable detection and quantification. The use of (tandem) mass spectrometry (MS), enabled the sensitive detection and quantification in order to better assess the co-occurrence of toxins. To the best of our knowledge this is a first report of a validated UPLC-MS/MS method for the simultaneous determination of beauvericin and the related enniatins, together with cereulide, in cereal and cereal-based food matrices such as wheat, maize, rice and pasta. A Waters Acquity UPLC system coupled to a Waters Quattro Premier XE™ Mass Spectrometer operating in ESI+ mode was employed. Sample pretreatment involved simple liquid extraction of the target toxins without any further clean-up step. The simple extraction, together with a fast chromatographic separation of only 7 min allowed substantial saving costs and time. The validation of the developed method was performed based on Commission Regulation No. 401/2006/EC and included determination of selectivity, repeatability, limit of detection (LOD), limit of quantification (LOQ), recovery and linearity. The obtained LODs ranged from 0.62 to 3.91 µg/kg and the LOQs from 1.24 to 7.83 µg/kg. The obtained RSD for repeatability was within 5 and 12% and the obtained RSD for intermediate precision was within 5 and 8%. The apparent recovery varied from 89 to 110%. For all compounds the extraction recovery varied between 85% and 105% in the different matrices. The highly sensitive and repeatable validated method was applied to a number of naturally contaminated samples allowing detection of sub-clinical doses of the toxins. Consequently, the influence of matrix (maize, wheat, rice and pasta) on the thermal stability of the target toxins under different conditions was investigated using the developed LC-MS/MS method.

Acknowledgements: this work was financially supported by the BOF Special Research Fund from Ghent University, GOA project No. 01G02213 and FWO mandate of Prof.dr. Andreja Rajkovic.

### 3.21. Development of a Multiplex Lateral Flow Immunoassay with Quantum Dots as Innovative Label

Foubert, A.; Beloglazova, N.; Rajkovic, A.; Sas, B.; Madder, A.; De Saeger, S.

Today, with the increased regulatory requirements in food safety the demand for rapid, sensitive and accurate methods to detect biological and chemical contaminants has increased. In particular, tests that can be completed within minutes would enable processors to take quick corrective actions when contaminants are detected, which is also the case for mycotoxins. Hence, rapid methods like the lateral flow immunoassay (LFIA) are rapid, user-friendly and sensitive on-site tests suitable for this purpose. Here, we present the development of a multi-mycotoxin LFIA system by using quantum dots (QD) as an innovative label. QDs, small semiconductor nanoparticles, are one of the most promising labels due to their unique spectral properties. They are characterized by a high fluorescence quantum yield, stability against photobleaching, and size-tunable absorption and emission bands. They allow simultaneous use of multiple QDs with different spectral characteristics (multiplexing). The stable photoluminescence makes QDs ideal nanoprobes for chemical, biomedical and therapeutic labeling and imaging.

In this work a multi-mycotoxin LFIA based on the use of green, red and orange-emitted QDs was developed and is able to detect four mycotoxins, *i.e.*, deoxynivalenol (DON), zearalenone (ZEN) and T2/HT2 in different matrices (barley and wheat). The test is based on an indirect competitive approach. First, the QDs were solubilized by coating them with polymer or silica, which also made bioconjugation with antibodies (Abs) possible. Next, the specific Abs were immobilized on the QDs by carbodiimide chemistry. The mycotoxin ovalbumin (OVA) conjugates (DON-OVA, ZEN-OVA and T2-OVA) were synthesized and immobilized as three test lines on the membrane. The test is completed within 15 min and there is no need for any mathematical or statistical processing of the obtained results. This detection method is a user-friendly and sensitive detection method with cut-offs (DON: 1000 µg/kg, ZEN: 80 µg/kg, T2/HT2: 80 µg/kg) according to EU legislation. Afterwards, the QD-based LFIA was also compared with a LFIA based on another, often used label, *i.e.*, gold nanoparticles. This showed that the QD-based LFIA was more sensitive and resulted in less use of antibody and antigen.

Acknowledgements: this work is financial supported by the BOF Special Research Fund from Ghent University, GOA project No. 01G02213.

### 3.22. Dried Blood Spots as a Powerful Tool for Individual Mycotoxin Exposure Analysis in Consumer’s Blood

Osteresch, B.; Cramer, B.; Humpf, H.-U.

Mycotoxins are toxic secondary metabolites of moulds that frequently occur in food. The intake varies depending on food contamination and consumption habits. Therefore, individual exposure to mycotoxins and their metabolites is difficult to evaluate when mean food contamination is used as calculation basis. However physiological samples like urine or blood can be analyzed to determine the exposition for each test person individually. In recent years dried blood spots (DBS) came more and more into focus for medical applications and sample collection in comparison to conventional vein puncture. Here, the DBS sample technique in combination with HPLC‑MS/MS detection is introduced for the quantitative assessment of mycotoxin exposure in humans.

For sample preparation, blood is collected on commercially available filter paper cards (Whatman 903 protein saver cards™), dried and extracted by an aqueous organic solvent. Then, an aliquot of the extraction solution is evaporated, reconstituted in mobile phase and injected into HPLC-MS/MS. The method has been validated concerning recovery, reproducibility, limit of detection and stability tests. Furthermore, effects on quantification like changes in hematocrit value and spotted blood volume have been investigated. For this, spiked blood samples were spotted and the ratio of detected concentrations in punched DBS were compared to whole DBS of known volume. As a result, altering hematocrit values and spotted blood volumes do not affect the quantification. In the same way the location of the sampling site for blood donation (fingertip or vein) caused no considerable issue.

The developed method provides sufficient mean recovery rates of 80%–120% and, for the majority of the included mycotoxins and metabolites, limits of quantitation in the lower pg/mL range. Storage tests show high stability for ochratoxin A for months. On the contrary compounds such as aflatoxins reveal strong degradation rates within weeks when stored in the darkness at room temperature. In a first study with coffee/noncoffee drinkers (*n* = 50) ochratoxin A could be found in all samples. In addition, both cohorts could be distinguished by the detection of the thermal degradation product 2’R‑ochratoxin A which is formed under coffee roasting conditions.

In conclusion, the developed HPLC-MS/MS method is a new, simple and sensitive way to estimate the mycotoxin exposition in consumer’s blood based on DBS.

### 3.23. Interaction between Fusarium Mycotoxins and Cytochrome P450 drug Metabolizing Enzymes/ABC Drug Transporters in a Porcine Animal Model

Schelstraete, W.; Devreese, M.; Van Bocxlaer, J.; Croubels, S.

The cytochrome P450 (CYP450) enzymes and ABC drug transporters in the intestine and liver play a major role in the pharmacokinetics of drugs and other xenobiotics. When drugs and food or feed are co-administered, this can lead to pharmacokinetic interactions due to inhibition or induction of CYP450 and ABC transporters by feed components. Consequently, a change in absorption, distribution, metabolism and/or excretion (ADME) of substrate drugs may take place. This has led to a number of clinically relevant changes in the pharmacodynamics of drugs. However, little is known about pharmacokinetic interactions between drug and food or feed contaminants. Mycotoxins are such contaminants produced by fungi and are the number one threat regarding chronic toxicity. *Fusarium* is one of the most important mycotoxigenic fungi genera and, consequently, this study will focus on mycotoxins produced by those species, namely deoxynivalenol, T-2 toxin, fumonisin B1 and zearalenone. For instance, our group has demonstrated an inhibition of hepatic CYP3A activity in pigs and the down-regulation of hepatic *CYP3A37*, *CYP1A4*, *CYP1A5* and *MRP2* in broiler chickens after exposure to T-2 toxin (Goossens *et al.* 2013; Osselaere *et al.* 2013). Supported by these previous results, it is the aim of our research to investigate the influence of oral exposure to *Fusarium* mycotoxins on the pharmacokinetic behavior of selected drugs in a porcine animal model.

To tackle this, *in vitro* and *in vivo* studies will be set up. A first goal is to geno- and phenotype key intestinal and hepatic CYP450 and drug transporters in pigs, in order to gain basic insight in the expression and activity of these proteins. Next, *in vitro* modulatory effects of *Fusarium* mycotoxins on these enzymes and transporters will be assessed, as well as the impact on mRNA expression and functional level in *in vivo* trials. These data will finally serve as decision criteria to select those *Fusarium* toxins with a significant impact, in order to conduct an *in vivo* trial to determine the effect of the mycotoxin(s) on the pharmacokinetics of substrate drugs.

Acknowledgements: this research is supported by the Special Research Fund (BOF DOC.2015.0075) from Ghent University.

Goossens, J.; De Bock, L.; Osselaere, A.; Verbrugghe, E.; Devreese, M.; Boussery, K.; Van Bocxlaer, J.; De Backer, P., Croubels, S. The mycotoxin T-2 inhibits hepatic cytochrome P4503A activity in pigs. *Food Chem. Toxicol.* **2013**, *57*, 54–56.

Osselaere, A.; Li, S.J.; De Bock, L.; Devreese, M.; Goossens, J.; Vandenbroucke, V.; Van Bocxlaer, J.; Boussery, K.; Pasmans, F.; Martel, A.; *et al*. Toxic effects of dietary exposure to T-2 toxin on intestinal and hepatic biotransformation enzymes and drug transporter systems in broiler chickens. *Food Chem. Toxicol.* **2013**, *55*, 150–155.

### 3.24. Presence of Deoxynivalenol in Wheat- and Corn-Based Products Produced in Serbia

Farkas, H.; Marosanovic, B.; Jaksic, M.; Cujic, S.; Marinkovic, D.

Deoxynivalenol (DON) is a natural-occurring mycotoxin mainly produced by *Fusarium graminearum*. It is also known as vomitoxin due to his strong emetic effects. Most often the compound is found in corn, but it is also found in other important crops such as wheat, oats, barley, rice, and other grains. Generally, the *Fusarium* species grow in moist, cool conditions and similarly invade crops under these more favorable conditions. The objective of this study was to investigate the presence of DON in corn- and wheat-based products produced in Serbia. Commercial samples were collected between January 2013 and December 2015 and analyzed by high performance liquid chromatography coupled with fluorescence detection (HPLC-FLD). Samples were prepared by immuno-affinity clean-up column. The method used for detection of the mycotoxins in wheat and corn - based products obtained a recovery rate from 92% to 99%, and limit of quantification was 60 µg/kg. During named period, we have analyzed 3765 samples for presence of DON. In 2013, 0.86% of analyzed wheat- and corn-based products were contaminated by DON, while in 2014 it was 2.63% and in 2015 23.5%. Concentration ranges were, retrospectively 75–498 µg/kg for 2013, 67–434 µg/kg for 2014 and 63–493 µg/kg for 2015. Maximum level for DON according to Commission Regulation (EC) No 1881/2006 in cereals intended for direct human consumption, cereal flour, bran and germ as end product marketed for direct human consumption is 750 µg/kg and for bread (including small bakery wares), pastries, biscuits, cereal snacks and breakfast cereals is 500 µg/kg, which has not been exceeded, but we noticed increased presence of DON in 2015. Despite the low contamination observed in wheat- and corn-based products, monitoring the presence of mycotoxins in foods is important to ensure safety.

### 3.25. Review of Principal Mycotoxins Occurrence in Food and Feed in Serbia during 2004–2014 Period

Udovicki, B.; Rajkovic, A.

This paper is a comprehensive review of relevant published investigations during a 10-year period (2004–2014) aimed at providing the necessary overview of occurrence of principal mycotoxins in food and feed in Serbia. This work has been done in the framework of the Horizon2020 project “MycoKey”. Mycotoxins, as secondary metabolites produced by several genera of fungi, are usually found in agricultural commodities and subsequently in animal products from animals that consumed such commodities as feed. Serbia is an agricultural country and grain production, especially wheat and maize, is an important economic factor as Serbia represents one of the largest maize producers and exporters in Europe. Cereals are an essential food for population in Serbia, having high social and nutritional relevance and to represent a food group with high risk for acute and chronic exposure to mycotoxins. In February–March 2013 several European countries, including Serbia, Croatia and Romania reported nationwide contamination of milk for human consumption (and possibly of derivative products) with aflatoxins. It was reported in March of the same year that feed originating from Serbia and imported in the Netherlands and Germany was contaminated. It was also reported in March that tests revealed contamination in milk produced by two Dutch farms. This large scale incident inspired many research efforts in the subsequent period. Out of 2595 samples tested in the period 2004–2014 75.7% were positive for aflatoxins, with 34.9% over the EU limits. It must be stated that most of positive samples are after year 2012 (after heavy drought in 2012). In that period 760 samples are tested with 70% of positive samples with 43.6% over EU limits. Out of 921 samples tested for DON 44.8% were positive, with 3.4% over the EU limits. Out of 490 samples tested for ZEA 34.7% were positive, with 5.7% over the EU limits. Out of 360 samples tested for FUMs 67.5% were positive, with only 0.3% over EU limits. Out of 363 samples tested for T-2/HT2 48.2 were positive, with 12.7% over EU regulation. Out of 483 samples tested for ochratoxin A 29.4% were positive, with 3.7% over the EU limits. Apart from the increase in the number of positive samples during years with favorable conditions for fungi growth and toxins production, in recent years that increase may also be attributed to more sensitive immunosorbent assays and HPLC/MS methods.

### 3.26. Analysis of 648 Feed Samples Sourced in Europe in 2015 for More than 380 Mycotoxins and Secondary Metabolites

Muccio, M.; Naehrer, K.; Kovalsky, P.; Krska, R.; Sulyok, M.

A total of 648 feed samples such as corn, wheat, barley, silage, as well as finished feed and others were screened for more than 380 mycotoxins and other secondary metabolites. The feed samples were collected in Europe in 2015 and analyzed with a multi-mycotoxin LC-MS/MS method at IFA-Tulln according to Vishwanath *et al.* (2009). The analytical method was transferred to a more sensitive mass spectrometer (QTrap^®^ 5500) and extended to cover more than 380 metabolites (Malachova *et al.* 2014; Streit *et al.* 2013). The accuracy of the method is monitored by regular participation in proficiency tests, which includes a separate testing scheme on “animal feed” (BIPEA, Gennevilliers, France).

On average 31 different metabolites were detected per sample. Seventy-seven percent of the samples tested positive for deoxynivalenol, 67% for nivalenol, 66% for zearalenone and 62% for deoxynivalenol-3-glucoside (average of positives 681, 71, 207, 108 µg/kg; max. 34,861, 5771, 6239, 2741 µg/kg). Thirty-eight percent of the samples were contaminated with fumonisins, 38% with A-trichothecenes and 81% with B trichothecenes (average of positives 241, 10, 689 µg/kg; max. 18,411; 557; 39,158 µg/kg).

The “emerging mycotoxins” emodin, enniatin B1 and beauvericin were found in over 81%; 79% and 56% of the samples analyzed respectively (average of positives 102, 45, 41 µg/kg; max. 2667; 662; 1601 µg/kg).

As the sensitivity of LC-MS/MS increased by 200-fold in the last 10 years, more mycotoxins and other secondary metabolites are detected per sample nowadays. In consequence, further data on the metabolic fate, mode of action, toxicity and interactions of mycotoxins are required to interpret the health risk. Nevertheless, *Fusarium* mycotoxins like deoxynivalenol, zearalenone and fumonisins are still among the most frequently occurring agriculturally relevant mycotoxins.

Malachova, A.; Sulyok, M.; Beltran, E.; Berthiller, F.; Krska, R. Optimization and validation of a quantitative liquid chromatography-tandem mass spectrometric method covering 295 bacterial and fungal metabolites including all regulated mycotoxins in four model food matrices. *J. Chromatogr. A* **2014**, *1362*, 145–156.

Streit, E.; Schwab, C.; Sulyok, M.; Naehrer, K.; Krska, R.; Schatzmayr, G. Multi-mycotoxin screening reveals the occurrence of 139 different secondary metabolites in feed and feed ingredients. *Toxins* **2013**, *5*, 504–523.

Vishwanath, V.; Sulyok, M.; Labuda, R.; Bicker, W.; Krska, R. Simultaneous determination of 186 fungal and bacterial metabolites in indoor matrices by liquid chromatography/tandem mass spectrometry. *Anal. Bioanal. Chem.* **2009**, *395*, 1355–1372.

### 3.27. Exploring the Effects of Gaseous Ozone (O_3_) and 1-Methylcyclopropene (1-MCP) Treatments on the Development of Penicillium Expansum and Patulin Production on Apple Fruits (cv. Granny Smith)

Testempasis, S.; Myresiotis, C.; Tanou, G.; Molassiotis, A.; Karaoglanidis, G.S.

“Blue mold’’ caused by *Penicillium expansum* is considered to be one of the most destructive postharvest diseases of apple fruit. The pathogen may lead to great quantitative losses while contributing to qualitative deterioration of apple products due to the production of a great variety of mycotoxins, such as patulin and citrinin that impose a risk for human health. Control of the disease is achieved by fungicide treatments. However, development of fungicide resistance and social concerns regarding pesticide residues, necessitate research for alternative control methods. The aim of this study was to evaluate the effect of 1-MCP (0.5 µL/L, 24 h, 0 °C) and ozone treatments (0.3 μL/L) on blue mold severity and patulin production on artificially inoculated apple fruits (cv. Granny Smith). In detail, artificially inoculated apple fruit, treated or not with 1-MCP, subjected for 2 and 4 months to cold storage (0 °C, RH > 95%) either in an O_3_ enriched atmosphere or in a conventional cold chamber. Results showed that disease severity was higher in both O_3_ and/or 1-MCP treated fruit, compared to the non-treated control fruit. To elucidate whether the increased susceptibility of apple fruit to ozone treatments was mediated by a predisposition of apple due to ozone applications, an additional experiment was conducted. 1-MCP treated apple fruit were exposed to ozone for 4 and 8 days prior the inoculation and incubated for 15 days in room temperature (20 °C, RH > 95%). Measurements of disease severity showed that the 1-MCP treated fruit, which had/had not previously been exposed for 4 and 8 days to O_3_, were more susceptible than the untreated fruit. In both experiments, patulin production was measured with High Performance Liquid Chromatography-Diode array detector (HPLC-DAD). 1-MCP-treated fruit that had been exposed to O_3_ for 8 days, showed 35 times higher patulin production than the untreated fruits. Similarly, 1-MCP treated fruit, stored in ozone chambers for 2 and 4 months, revealed higher patulin concentration than the untreated fruit. Such results emphasize that 1-MCP and O_3_ treatments not only do not contribute to the control of the disease but in addition, appear to be directly related to an increased patulin production. Further research is required to explore the molecular basis of this increase in blue mold incidence and patulin production in 1-MCP and O_3_-treated apple fruit.

### 3.28. Biomarkers for Exposure of Mycotoxins in Pigs and Broiler Chickens

Lauwers, M.; De Baere, S.; Letor, B.; De Saeger, S.; Croubels, S.; Devreese, M.

Poultry and pigs are highly exposed to mycotoxins due to their cereal based diet. Acute and chronic exposure to mycotoxin contaminated feed can cause deleterious effects on the performance and wellbeing of the animal and leads to economic losses. Therefore, it is of great importance to assess mycotoxin exposure in livestock and to correlate this with the health status of the flock or herd. This can be done by using biomarkers as indicator of exposure. Typical biomarkers for exposure are parent compounds and their phase I and II metabolites, which can be detected in biological matrices, such as plasma and excreta.

Besides exposure assessment, alleviating the effects of mycotoxins on animals is of critical importance. Mycotoxin detoxifying agents, or mycotoxin detoxifiers, are feed additives which aim to bind (mycotoxin binders) or to alter the chemical structure (mycotoxin modifier) of the mycotoxin in the gastro-intestinal tract of the animal, thereby diminishing the exposure of animals to mycotoxins. The European Food Safety Authority (EFSA) has stated that the efficacy of these compounds should be evaluated *in vivo* by analysing the concentration of a suited biomarker, *i.e.*, the parent compound and/or the metabolites, in biological matrices.

The first objective of this PhD research is to develop an ultra-high performance liquid chromatography coupled to HR-MS (type Synapt G2-Si HDMS) screening method to determine the mycotoxins aflatoxins, deoxynivalenol, T-2 toxin, fumonisins, zearalenone and enniatins and their major phase I and II metabolites in plasma, urine and faeces of pigs and plasma and excreta of poultry. Optimization of the LC parameters (column, gradient, solvents, temperature, *etc.*) led to the successful separation and detection of 24 mycotoxin analytical standards. Secondly, feed and selected feedstuffs will be screened for mycotoxin contamination. In the future, toxicokinetic studies of the most prevalent mycotoxins will be performed in pigs and broiler chickens to evaluate species dependent differences in absorption, distribution, metabolisation and excretion (ADME) processes of these mycotoxins in both animal species. Furthermore, the most appropriate biomarkers of exposure will be identified for each mycotoxin in both animal species.

Next, the effect of candidate mycotoxin detoxifiers on the toxicokinetic behavior and selected biomarkers of the mycotoxins will be evaluated.

Finally, the most potent mycotoxin detoxifier(s) will be retained and assessed in long-term feeding trials where a mycotoxin contaminated diet is fed to the animals with and without detoxifier.

### 3.29. MIP Loaded Porous Scaffolds as SPE Sorbent for Ergot Alkaloid Analysis

De Middeleer, G.; Dubruel, P.; De Saeger, S.

Mycotoxins are naturally occurring contaminants in food and feed which are produced by various fungal species. Although these secondary metabolites are only present in ppb-ppt concentrations, they can be toxic to humans and animals. Within the group of mycotoxins, ergot alkaloids are produced by *Claviceps* species and they occur mostly in cereal based products. Since infected crops can be processed for consumption, the economic consequences of such contaminations should not be underestimated. Therefore, rapid, sensitive and accurate analysis is obligatory to control and secure food and feed safety. In general, mycotoxin analysis includes rapid screening and confirmatory methods, mostly focussing on a multi-analyte approach. Therefore, selective recognition elements are required such as antibodies or molecularly imprinted polymers (MIP) which can bind with different target analytes. Since antibodies suffer from several disadvantages such as low stability and high production costs, this research aims to use MIP for the development of a solid phase phase extraction (SPE) application prior to ergot alkaloid LC-MS/MS analysis. This application needs to cover the extraction of the six major ergot alkaloids and their corresponding epimers.

Sub-micrometer sized MIP for ergot alkaloids analysis have been produced by precipitation polymerization. Equilibrium experiments indicated that MIP particles bind higher amounts of metergoline template molecules compared to non-imprinted particles. In addition, different conditions were tested to evaluate binding characteristics of the MIP more specifically, to study the binding of a mixture of the six major ergot alkaloids and their corresponding epimers through recovery experiments. Since the final application implies MIP to be immobilized onto poly-ε-caprolactone (PCL) structures, the particles were deposed by means of Pluronic^®^ F127 bismethacrylate hydrogel building blocks. The optimization of the immobilization protocol and selection of the optimal hydrogel concentration were first examined on 2D PCL-spincoated glass plates. These immobilization experiments and sol-gel tests have shown that 7.5% and 10% of hydrogel resulted in successful particle immobilization and respectively an 87.4% and 83.3% gel-fraction of the corresponding hydrogel network. Second, MIP particles need to be immobilized on 3D structures to meet the intended SPE application. Therefore, the Bioplotter^TM^ technology was used to produce 3D PCL scaffolds which are characterized by micrometer sized interconnective pores. The immobilization of MIP onto these scaffolds was successful as shown by SEM analysis. In a next step, the functionality of the MIP particles onto the 3D structures was investigated to examine whether the MIP binding capacity is still sufficient after immobilization on 3D scaffolds.

Acknowledgements: the authors thank the agency for Innovation by Science and Technology (IWT) for the financial support.

### 3.30. Elucidation of Bikaverin Biosynthesis in Fusarium Fujikuroi by Genetic Engineering, High Resolution Mass Spectrometry and NMR

Arndt, B.; Studt, L.; Wiemann, P.; Tudzynski, B.; Köhler, J.; Krug, I.; Humpf, H.-U.

In this study, we have identified an unknown metabolite in *Fusarium fujikuroi*, an ascomycetous fungus normally infecting rice plants resulting in yield losses. This fungus gained attention due to produced phytohormones, gibberellines, which constitute a virulence factor of the fungus but are nowadays also used as plant growth regulators in agriculture. Since the genome of *F. fujikuroi* is fully sequenced, the identified metabolite could be assigned to the corresponding gene cluster through genomic engineering, showing that the substance is dependent on the bikaverin gene cluster. Although all genes for the biosynthesis of the PKS-derived pigment are known, only two bikaverin precursors, nor-bikaverin and pre-bikaverin, are established so far.

With the help of genomic engineering and high-performance liquid chromatography (HPLC) coupled to high resolution mass spectrometry (HRMS) followed by isolation and detailed structure elucidation, the new substance could be designated as an unknown bikaverin precursor, missing two methyl- and one hydroxy group, hence named oxo-pre-bikaverin. To decipher the whole bikaverin biosynthetic pathway and to overcome negative regulation circuits, the structural cluster genes *BIK2* and *BIK3* were overexpressed independently in the ΔΔ*bik2/bik3* + OE::*BIK1* mutant background by using strong constitutive promoters. With the help of the software MZmine 2, the metabolite spectra of the created mutants were compared, revealing further possible intermediates.

To analyze the potential cytotoxic properties of this new compound, we compared the effects of bikaverin and the new intermediate on Hep G2 cells using a cell proliferation and cytotoxicity assay (CCK-8). Oxo-pre-bikaverin (1 nM–100 µM) showed no cytotoxic effect but a concentration dependent increase of cell viability by up to 62%. Treatment with bikaverin showed no cytotoxic effects.

Acknowledgements: financial support by the DFG and the NRW Graduate School of Chemistry is gratefully acknowledged.

### 3.31. Occurrence and Quantitation of the Non-Cytotoxic Tenuazonic Acid Isomer Allo-Tenuazonic Acid in Tomato Products

Hickert, S.; Krug, I.; Cramer, B.; Humpf, H.-U.

Tenuazonic acid (TeA) is a mycotoxin mostly produced by fungi of the genus *Alternaria* on various foodstuff. *Allo-*tenuazonic acid (*allo*-TeA) is a known acid and base catalysed degradation product of TeA. Routine methods described in literature for the quantitative determination of TeA in food samples do not distinguish between TeA and *allo-*TeA as the chromatographic separation of both diastereomers is challenging. The separation of both compounds using inorganic salts as mobile phase additives is described in literature—but these chromatographic approaches are not compatible to HPLC-MS/MS as these additives are not volatile. The scope of this work was to develop a chromatographic method for the separate detection of both diastereomers using an HPLC-MS/MS compatible solvent system and its application to food samples. Furthermore, *allo*TeA should be isolated and tested against TeA for its cytotoxicity in cell culture.

*Allo-*TeA and TeA could be separated on a preparative scale using RP-amide material. TeA and *allo*-TeA were obtained in isomeric purities >98%. Application of *allo*-TeA to HT-29 cells revealed no cytotoxicity (10–800 µM) while TeA showed toxic effects starting at 250 µM. A chromatographic method separating both isomers on an analytical scale using hypercarb material and a binary gradient consisting of methanol and water, both containing 1% FA and 10 mM NH_4_OAc, was successfully developed. Using a previously synthesized ^13^C_2_-labeled standard of TeA and *allo*TeA [4], 20 tomato samples were analyzed for their TeA and *allo*TeA levels, showing that all 20 tomato products contained both epimers. TeA was found in concentrations from 5.3–540 µg/kg (average: 110 µg/kg) and *allo-*TeA in a range of 1.4–270 µg/kg. On average, *allo-*TeA represents 27% of the sum of both epimers. *Allo-*TeA can be found in small amounts (<5%) when *Alternaria alternata* is cultivated on tomato puree.

As *allo-*TeA shows differences in toxicity compared to TeA and constitutes a major portion of the sum of both epimers, approaches quantifying both as a sum parameter might overestimate the risk to consumers. The co-occurrence of both isomers may have implications on future legal limits in the European Union.

### 3.32. Piperazine and Benzodiazepine-Derived Metabolites from Penicillium Aurantiogriseum

Kalinina, S.; Hickert, S.; Cramer, B.; Humpf, H.-U.

Fungi are considered as major plant and insect pathogens as well as important agents of disease in vertebrates. They produce a multitude of low-molecular-weight compounds known as mycotoxins, which are toxic in low concentrations, causing acute and chronic diseases. Additionally, around 25% of crops worldwide are contaminated by molds and affected by mycotoxins, and the estimated loss extends to billions of dollars. Among others *Penicillium* species represent important toxigenic fungi, which are often found in food products and the range of mycotoxin classes produced by these fungi is much broader than that of any other genus. *Penicillium* toxins often affect liver and kidney function of vertebrates and are represented mainly by structurally diverse compounds such as penitrems, roquefortines, patulin, citrinin, griseofulvin, auranthine, PR-toxin and others. Nevertheless, some species of these genera are not well characterized and can produce several unknown toxic food contaminants. In the course of our study of toxins from *Penicillium* species in food, piperazine- and benzodiazepine-derived toxins from *P. aurantiogriseum* were successfully isolated. For that purpose, fungal cultures were cultivated on Czapek Dox Agar in the dark at 22 °C. After three weeks of cultivation fungal cultures were extracted with ethyl acetate followed by separation of compounds using flash chromatography (reversed-phase column SNAP KP-C18-HS) with an acetonitrile/water gradient. Final purification of piperazine and benzodiazepine-derived toxins was carried out with normal-phase flash chromatography using a gradient of cyclohexane/ethyl acetate. The structure of the isolated compounds was elucidated by NMR and HPLC-MS experiments. Substantial toxicity of the isolated compounds in cell culture experiment encourages further method development for their detection and quantification in food.

### 3.33. Monitoring Chemical Contaminants and Residues in Insects: Focus on Mycotoxins

Kowalski, E.; Wauters, J.; Croubels, S.; Claes, J.; Vanhaecke, L.

A growing world population together with ecological as well as economic concerns related to livestock industry enhances the quest towards alternative protein sources of which insects are acknowledged to have great potential. The new European Novel Food legislation (Regulation 2015/2283) requires that, before 1 January 2018 for all (products of) insects for human consumption, an application for authorization must be submitted for authorizing the placing on the market of these products. Until that date, the current Belgian tolerance of 10 insect species continues to apply. In this Novel Food regulation scientific evidence of the safety for human health must be demonstrated.

In the advice of the Federal Agency for the Safety of the Food chain (FASFC) and Superior Health Council (SHC), the potential microbiological, chemical, and physical hazards related to the consumption of edible insects, is questioned. In an attempt to guarantee health-safe end products, we aim to map and monitor the relevant (organic) chemical contaminants and residues. To achieve the latter purpose, the development of a broad-range analysis method, specific for insect tissues as well as their feed/substrates is mandatory.

The compounds of interest include at least 25 pesticides (herbicides and insecticides), 29 relevant veterinary drugs and coccidiostats, a bacterial toxin and a total of 25 mycotoxins. The mycotoxins originate from different fungal genera, *i.e.*, *Aspergillus, Penicillium, Fusarium* and *Alternaria.* For separation and detection, ultra-high performance liquid chromatography coupled to quadrupole Orbitrap high-resolution mass spectrometry (UHPLC-Q-Orbitrap^TM^-HRMS) is the platform of choice. Optimization of the parameters of both the LC (gradient, column, solvents, temperature, *etc.*) and MS (gases, temperature, voltage, *etc.*) resulted in a short method (10 min) that proved successful for the separation and detection of the 80 analytical standards. Assisted by a fractional factorial design, generic extraction protocols for several insect species (including *Hermetica illucens*, *Tenebrio mollitor*, *Locusta migratoria* and *Acheta domesticus)* and their potential substrates (including wheat-bran, grass, waste products (supermarket), dry cat food, chicken blood and faeces) are currently under development. Next, the analytical methods will be validated and applied for the targeted and untargeted analysis of the specified chemical contaminants and residues in edible insects and their substrates.

### 3.34. Comparative Analysis of Fusariotoxins Occurrence in Wheat, Barley and Corn Grain

Laurain, J.; Nacer Khodja, E.; Marengue, E.

Feed raw materials show different fusariotoxins occurrence depending on the type of culture, as shown by the Scientific Cooperation (SCOOP) survey in 2003. The aim of this study is to identify the occurrence of fusariotoxins in the three main cereals used by the feed industry: wheat, barley and corn grain. This study uses the ‘Laboratoire Public Conseil, Expertise et Analyse’ (LABOCEA) database composed of chromatography analyses run with LC-MS/MS from 2013 to 2015. Twenty-four fusariotoxins are tested in each analysis. The percentage of positive samples (>LOQ) and the median level of contamination (ppb) per mycotoxin are the two main criteria used. In order to avoid any geographical interaction, samples from one restricted area only (France) are considered: wheat (*n* = 274), barley (*n* = 104) and corn grain (*n* = 336). Data show that corn grain is more poly-contaminated than wheat and barley (per sample, on average seven fusariotoxins are positive for corn grain, two for wheat and three for barley). As a consequence, the percentage of positive samples per mycotoxin is more important in corn grain than for other cereals. Deoxynivalenol (DON) is the most frequent fusariotoxin (>90% of positive) in all cereals, but its median level of contamination is far higher for corn grain (740 ppb) than for wheat (215 ppb) and for barley (75 ppb). The levels of 15-*O*-acetyl DON, zearalenone (ZEA) and fumonisins are also significantly higher for corn grain (153; 135 and 345 ppb, respectively) than for wheat (15, 25 and 50 ppb, respectively) and barley (20, 25 and 30 ppb, respectively). Focusing only on straw cereals, wheat shows higher median contamination in DON, T-2 toxin and fumonisins (215, 35 and 50 ppb respectively) than barley (75, 10 and 30 ppb respectively), whereas barley is more often contaminated (% of positive samples) in DON acetylated forms (40.4% in 15-*O*-acetyl and 17.3% in 3-*O*-acetyl DON) than wheat (5.8% in 15-*O*-acetyl and 16.4% in 3-*O*-acetyl DON). The different cropping parameters (time of harvest, use of fungicide, *etc.*) of corn could explain the important differences in fusariotoxins occurrence compared to straw cereals. *Fusarium* strains have different developments between wheat and barley, which may explain the variable fusariotoxins occurrence.

### 3.35. Comparative Analysis of Fusariotoxins Occurrence in Different Types of Corn Materials

Laurain, J.; Nacer Khodja, E.; Marengue, E.

Feed raw materials show different profiles of mycotoxin contamination depending on the type of culture and storage conditions. Corn is known as a major source of mycotoxins and particularly of fusariotoxins as described in 2003 by SCOOP survey. The aim of the present study is to identify the occurrence of fusariotoxins in different types of corn materials: dry corn grain, humid corn grain (silage corn grain) and corn silage (fermented full corn plant). This study uses the LABOCEA database composed of chromatography analyses run with LC-MS/MS from 2013 to 2015. Twenty-four fusariotoxins are tested in each analysis. The percentage of positive samples (>LOQ) and the median level of contamination (ppb) per mycotoxin are the two main criteria used. In order to avoid any geographical interaction, samples from one restricted area only (France) are considered: dry corn grain (*n* = 337), humid corn grain (*n* = 119) and corn silage (*n* = 557). Data show that all types of corn materials are poly-contaminated with fusariotoxins (per sample, on average seven fusariotoxins are positive) and that deoxynivalenol (DON) is the most frequent (>90% of positive) and the most present (median value > 600 ppb) fusariotoxin. The profiles of fusariotoxins (% of positive samples) are very similar between corn raw materials whereas the level of median contamination depends on the type of corn material. Corn silages have higher DON median level of contamination (1090 ppb) than humid corn grains (980 ppb) than dry corn grains (720 ppb). Deoxynivalenol acetylated forms (15-*O*-acetyl and 3-*O*-acetyl DON) contaminations are similar for all types of corn materials. Nevertheless, nivalenol (NIV) median level in corn silage is 4 times higher than in corn grain (290 ppb *vs*. 68 ppb, respectively). On the contrary dry corn grains have higher median sum of fumonisins (320 ppb) than corn silage (40 ppb) and humid corn (68 ppb). Zearalenone (ZEA) occurrence is similar in all types of corn materials with contamination levels far lower than for DON (median level: <200 ppb for ZEA; >700 ppb for DON). Regarding type-A trichothecenes the profiles of contamination are equivalent for all types of corn materials apart for one metabolite (monoacetoxyscirpenol, MAS) which is more often present (% positive samples) in corn silage (66.4%) than in humid corn grain (32.8%) and in dry corn grain (11%). Some parameters like time of harvest and type of preservation may explain the variable profile of fusariotoxins among corn materials.

### 3.36. Interactions between Mycotoxins and the Rumen, Their Possible Toxicological Effects on the Gastrointestinal Tract and Their Intestinal Absorption in Dairy Cattle: An in Vitro Approach

Debevere, S.; Devreese, M.; De Baere, S.; De Saeger, S.; Haesaert, G.; Fievez, V.; Croubels, S.

Mycotoxins are more and more associated with subclinical health problems for high productive dairy cows reflected by vague and non-specific symptoms and periodic decrease in milk production. Indeed, the risk of mycotoxin contamination of dairy diets is high since the main components such as corn, grass silage and small grain cereals are susceptible to infection with toxigenic fungi. Moreover, considering the wide diversity of toxigenic fungal species on crops and the ability of several fungi to produce more than one mycotoxin, a multiple contamination can be expected. Hence, dairy cows are exposed to various mycotoxins which may lead to depletion of the detoxifying capacity of the microbiota in the rumen. To date more and more dairy farmers and veterinarians are concerned about the impact of mycotoxin on the health and performance of dairy cows. Therefore, research about this topic is needed.

This multidisciplinary doctoral research will elucidate the degradation of individual mycotoxins and relevant mycotoxin combinations in the rumen, the interactions between mycotoxins and the rumen function as well as the possible toxicological effects of mycotoxins on the gastrointestinal tract and their intestinal absorption. Mycotoxins that contaminate corn silage, the most important part of the diets of dairy cattle, will be studied by means of *in vitro* rumen simulations (static and continuous fermentation system). Also varying rumen conditions will be included (e.g., varying dietary ratios roughage/concentrate up to circumstances of subacute acidosis) as they may influence mycotoxin degradation. Ultra-high performance liquid chromatography coupled to high-resolution mass spectrometry (UHPLC-HR-MS, type Synapt G2-Si HDMS) will be used to identify and quantify mycotoxins and their metabolites in the rumen contents. Intestinal cytotoxicity and absorption of mycotoxins will be determined by means of relevant cell cultures. In addition, the efficacy of mycotoxin binders will be tested at the level of rumen metabolism, cytotoxicity and intestinal absorption.

Acknowledgements: this project is co-financed by Flanders Innovation & Entrepreneurship (VLAIO).

### 3.37. Beyond Aflatoxins: MS-Based Metabolomics Approaches to Investigate Known and Novel Fungal Secondary Metabolites in Aspergillus Flavus

Diana Di Mavungu, J.; Arroyo-Manzanares, N.; Uka, V.; Zheng, H.; Cary, J.W.; Ehrlich, K.C.; Bhatnagar, D.; Calvo, A.; Vanhaecke, L.; De Saeger, S.

The *Aspergillus flavus* metabolome is of considerable interest because *A. flavus* produces the secondary metabolites aflatoxins, the most toxic and carcinogenic compounds known from fungi that occur world-wide as contaminants of foods and animal feeds. Examination of the fungal genome revealed 55 gene clusters predicted to be involved in secondary metabolite biosynthesis, suggesting that *A. flavus* is capable of producing many more potentially significant metabolites than have thus far been reported.

To identify the metabolites produced by these yet unexplored gene clusters and the enzymatic steps involved in their biosynthetic pathways, we established a strategy based on the metabolic profiling of extracts of *A. flavus* wild-type and mutant strains, followed by the structural elucidation of differentially expressed metabolites. To date, we have closely examined metabolite production by wild type *A. flavus* and mutants in biosynthetic genes in clusters 23, 27 and 39. Our results show that cluster 27, with close similarity to the aflatoxin cluster, encodes a polyketide synthase (PKS) producing four anthraquinone metabolites including asparasone A, a compound previously identified in *A. parasiticus*. The hybrid PKS-nonribosomal peptide synthetase (NRPS) of cluster 23 is responsible for the production of a class of 2-pyridone compounds, the leporins, while the PKS of cluster 39 produces 4,7 didesmethyl-siderin, a precursor that is converted to a set of methylated and hydroxylated coumarin derivatives involved in the formation of aflavarin and other previously unidentified C3-C8’ linked bicoumarins.

In this presentation, the potential of high resolution mass spectrometry in both the structural confirmation and *de novo* identification of fungal secondary metabolites will be demonstrated using data from the above mentioned *A. flavus* gene clusters studies. In particular, the opportunity offered by new developments in mass spectrometry instrumentation and data processing tools for improving the current state of metabolite identification strategies will be discussed.

### 3.38. Development of an Immunochemical Test for Mycotoxin Detection Using Luminescent Nanobiolabel

Goftman, V.; Goryacheva, I.; De Saeger, S.

For the successful development of sensitive and reproducible immunoassay methods, the researcher has to settle two tasks: exploiting highly efficient signal-transduction labels and adopting simple, sensitive signal-transduction methods. In this regard, luminescent sensing provides fast, sensitive, reliable and reproducible detection of the target molecules. The qualitative characteristics of luminescent methods strongly depend on the label used. Quantum dots (QDs) have shown great potential as luminescent nanobiolabels because of their unique properties: nanoscale size, broad excitation spectra for multicolor imaging, robust, narrow-band emission and versatility in surface modification. First used for bioimaging, QDs have later become a useful tool for immunoassay in traditional microtiter-plate format (fluorescent-linked immunosorbent assay, FLISA), sensors and rapid tests.

Our research is focused on the development of a stable luminescent nanobiolabel based on QDs for the sensitive detection of mycotoxins, which are toxic secondary metabolites produced by molds. To this end, first, an efficient synthesis of QDs with different emission colors was carried out. Second, silanization was applied as a modification technique to obtain functional biolabels. In particular, the nanolabel surface was modified with diverse functional groups (amino-, carboxyl-, epoxy-, mercapto-), as such controlling its charge. High buffer stability and low non-specific sorption was achieved by using PEG-derivate silanes.

Third, bioconjugation with secondary rabbit anti-mouse antibodies (IgG) was performed in order to prepare the fluorescent label for indirect immunoassay. An indirect format of immunoassay was chosen in order to increase the sensitivity of the developed method. Moreover, the synthesized conjugate of QDs and IgG is a universal reagent for all monoclonal mouse antibodies, which opens an opportunity to use it for the detection of different analytes, including multiplex detection of different antigens.

Finally, the synthesized luminescent labels were successfully applied in FLISA for the determination of deoxynivalenol (DON) mycotoxin. To decrease time of analysis and to obtain a rapid system for on-site detection, a novel polyethylene frit-based immunoassay for DON determination was also developed. Both methods showed good precision, but the frit-based immunoassay allowed a decrease in IC_50_ by a factor of 20 (IC_50_ FLISA was 473 ng/mL, while IC_50_ for frit-based immunoassay was 20 ng/mL). The total assay time for FLISA was 16 h (using laboratory equipment at each stage), while for frit-based rapid test—only 1 h. Additional advantage of frit-based assay is the possibility to work in a non-laboratory environment (in the case of availability of a handheld reader).

### 3.39. Impact of Chronic Multi-Mycotoxin Exposure in Europe on Cancer Incidence: a Basis to Develop Future Public Health Strategies

De Ruyck, K.; Heyndrickx, E.; De Boevre, M.; Huybrechts, I.; De Saeger, S.

Mycotoxins are fungal toxins, estimated by the Food and Agricultural Organization (FAO) to contaminate 25% of the world’s most frequently consumed foods and feeds. Several fungi may co-occur on crops, resulting in co-occurrence of multiple mycotoxins. Given the ubiquity of many fungi worldwide, an urgent need exists for a coordinated international response to the problem of dietary mycotoxins.

In terms of chronic toxicity, mycotoxins are estimated to be the most hazardous food contaminants. The International Agency for Research on Cancer (IARC) identifies aflatoxins B1, G1, and M1 as sufficiently evident carcinogens, while other mycotoxins are possibly or probably carcinogenic (e.g., ochratoxin A and fumonisins). Consumed with food, some mycotoxins may be absorbed by the blood stream to most commonly affect the liver, where they are metabolized, though not always inactivated. Further down the gastro-intestinal tract, other less bio-available mycotoxins may cause significant interactions with colon cancer cells.

Though the current ‘gold standard’ for estimating dietary mycotoxin exposure consists of food-based mycotoxin occurrence data cross-referenced against food consumption data, this estimate may be considered the ‘external exposure’ quantity. Alternatively, measurements made through physiological tissues may be considered representative of an individual’s ‘internal exposure’ to mycotoxins.

A previously published UHPLC-MS/MS method for simultaneous determination of multiple mycotoxins has recently been augmented with methods for the determination of several major mycotoxin metabolites. Particularly, glucuronides of deoxynivalenol, HT-2 toxin, and zearalenone may be present in the blood or urine. Measurement of these metabolites may provide useful insights toward characterizing dietary mycotoxin exposure.

Preliminary analyses of combined data from EFSA and the European Food Consumption Validation (EFCOVAL) Project reported unexpectedly high external exposures, some above upper tolerance levels (unpublished data). This dataset will have been validated through UHPLC-MS/MS quantification of mycotoxin ‘exposure biomarkers’ in serum and urine, by the time of this conference.

This is the first large-scale cohort study investigating the effect of multi-mycotoxin intakes on incidence of specific forms of cancer. The resulting mycotoxin databases will be instrumental for further characterizing the health effects of real exposure.

### 3.40. Identification of Mercapturic Acid Conjugates of Deoxynivalenol in Cereals

Stanic, A.; Uhlig, S.; Rise, F.; Hofgaard, I.S.; Miles, C.O.

We recently synthesized and characterized the L-cysteine and glutathione (GSH) conjugates of 4-deoxynivalenol (DON). The amino acid and the tripeptide were shown to be linked to DON via conjugation of the 9,10-double bond or the 12,13-epoxide. We also reacted DON with γ-glutamyl-cysteine, cysteinyl-glycine and *N*-acetyl cysteine (“mercapturic acid”) and characterized the product mixtures using liquid chromatography coupled to low- and high-resolution mass spectrometry. Extracts from cereal samples (*n* = 40, oats and spring wheat), which were naturally contaminated with DON at a concentration of 1.1 to 11 mg/kg, were screened for the presence of DON-GSH conjugates and their breakdown products. Both C-10 and C-13 linked DON-GSH conjugates were detected in two oats samples. Furthermore, two *N*-acetyl cysteine conjugated DON isomers were detected in 15 samples (both grain species). The LCMS-data (retention time, HRMS^2^) suggested that the *N*-acetyl cysteine moiety in the two isomers was likewise linked to DON via C-10 and C-13 conjugation. Our data: (1) confirms that GSH-conjugation of DON occurs in plants; (2) shows that conjugation occurs both at C-10 and C-13, and; (3) suggests that DON-mercapturate could be a major breakdown product of such DON-GSH conjugates in planta.

### 3.41. Occurrence of Fusarium Mycotoxins and Their Modified Forms in Major Cereal Crops and Their Processed Product (ogi) from Nigeria

Chilaka, C.A.; De Boevre, M.; Atanda, O.; De Saeger, S.

In Nigeria, maize, sorghum and millet rank as the most important crops. They are prepared in several forms and consumed on daily basis. These crops are prone to fungi infestation, and as a result may be contaminated with toxic secondary metabolites. Despite the health hazards caused by these mycotoxins, Nigeria has devoted minimal attention to these arrays of toxins especially those produced by *Fusarium* fungi. *Fusarium* mycotoxins such as fumonisins, zearalenone and trichothecenes are the most common mycotoxins contaminating a wide range of crops, especially cereals and cereal-based foods worldwide. They have been reported to cause a variety of toxic effects in animals as well as in humans.

A great concern regarding *Fusarium* mycotoxin contamination of foods is the error of underestimation of mycotoxin levels as a result of modification of these mycotoxins. Modification can be conjugated by plants, animal or fungi, matrix-related or occurring during food processing. They easily escape routine mycotoxin detection methods, and when ingested, may subsequently hydrolyse to free toxins in the digestive tract of organisms. To date, a detailed study on the occurrence of *Fusarium* mycotoxins and their modified forms remains a knowledge gap in Nigeria. We aim to survey and investigate the occurrence of *Fusarium* mycotoxins and their modified forms in Nigerian maize, sorghum, millet and their processed product (*ogi*) available in the market from four agro-ecological zones in the country.

A total of 363 samples (maize *n* = 136, sorghum *n* = 110, millet *n* = 87 and *ogi* *n* = 30) were sampled between September 2015 and October 2015. Analytical methods using LC-MS/MS have been developed and validated for the different matrices in this study. The expected results will contribute to the formulation of a national food safety action plan.

Acknowledgements: authors would like to acknowledge Ghent University Special Research Fund (BOF-01W01014) for funding first author’s doctoral studies.

### 3.42. Unraveling the Detoxification Mechanism of Deoxynivalenol in Aphids Using Targeted and Untargeted Analysis

Arroyo-Manzanares, N.; De Boevre, M.; de Zutter, N.; Smagghe, G.; Haesaert, G.; Audenaert, K.; De Saeger, S.

Mycotoxins are toxic, low-molecular-weight, secondary metabolites produced by fungi. Although they are produced by several fungal genera their function often remains elusive. One exception is the trichothecene deoxynivalenol (DON) which is a well-known virulence factor during the infection process of *Fusarium graminearum* helping the fungus to colonize wheat ears. *F. graminearum* is not the only pathogen present on wheat ears: grain aphids (*Sitobion avenae*) co-occur with *F. graminearum* and the aphids come in contact with DON by ingesting plant phloem of *F. graminearum* infected ears. This tripartite interaction between grain aphids, *Fusarium* and wheat was investigated in current study.

*In vitro* studies revealed that grain aphids can thrive well on artificial diets amended with high concentrations of DON (up to 100 ppm) while pea aphids (*Acyrthosiphon pisum*), which are insects infecting vegetables and never encounter DON, are susceptible to DON. This result suggests that grain aphids have adapted to living in the presence of *F. graminearum* and its mycotoxin DON. We are the first research group providing evidence for a detoxification mechanism in grain aphids enabling them to detoxify DON.

The presented study reports the targeted and untargeted analysis of *Sitobion avenae* to unravel the detoxification mechanism of DON in these insects. Via extensive analyses the authors were able to state that *grain aphids* are more efficient in converting DON into glycosylated forms than pea aphids. For the first time, DON, deoxynivalenol-3-glucoside (DON3-G) and three isomers of DON-diglucoside were identified in *Sitobion avenae*. DON and DON3-G were quantified, and more DON-3G was present in *Sitobion avenae* compared to DON (*p* < 0.001). In addition, DON-diglucosides were identified for the first time, but could not be quantified as no reference standards were available.

This report points out the analytical methods used and the identification of the modified DON-forms.

### 3.43. Multidetection of Urinary Ochratoxin A, Deoxynivalenol and Its Metabolites: A Pilot Time-Course Study and Risk Assessment in Catalonia (Spain)

Vidal, A.; Cano-Sancho, G.; Marín, S.; Ramos, A.J.; Sanchis, V.

The prevalence of two main mycotoxins, ochratoxin A (OTA) and deoxynivalenol (DON), is widespread in cereal-based foodstuffs marketed in Europe. The objective of this study was to assess the urinary and plasma concentrations of OTA, ochratoxin α (OTα), DON, deoxynivalenol-3-glucoside (DON-3-glucoside), deoxynivalenol-3-glucuronide (DON-3-glucuronide), 3-acetyldeoxynivalenol (3-ADON) and de-epoxy-deoxynivalenol (DOM-1) in a preliminary follow-up trial in Catalonia (Spain). Urine and plasma mycotoxin levels and food dietary intake were prospectively monitored in a group of volunteers throughout a restriction period followed by a free-diet period. The results showed that urinary OTA, DON and its metabolites were detected in most of samples, displaying moderate reductions after the restriction period, and subsequently recovering the background levels. Despite the restriction period, some DON metabolites, such as 3-ADON or DOM-1, were found in most of urine samples, placing other alternative sources of exposure under suspicion. DON and DON-3-glucuronide were significantly associated with consumption of bread (*r* = 0.362, *p* < 0.001) and pastries (*r* = 0.239, *p* < 0.01), while OTA was only associated with consumption of wine and breakfast cereals. The urinary levels of OTA were significantly correlated with plasmatic levels of OTA and OTα, supporting the results from urine that allows the simultaneous determination of OTA and DON forms. Also it is more convenient to enroll donors in large-scale studies. The results also showed that the high exposure to DON could be held throughout the time by the same person, exceeding the total daily intakes (TDIs) systematically instead of eventually. The estimates of OTA exposure through urine were largely higher than those obtained with the dietary approach. The background levels found in urine revealed that the exposure to DON and OTA could be of concern for the Catalonian population, thus, further studies applying this biomonitoring methodology in a larger sample of population are needed to accurately characterize the human health risks at population level.

### 3.44. Mycotoxin Contamination in Sugar Cane Grass and Juice: First Report on Multi-Toxins Detection and Exposure Assessment in Humans

Abdallah, M.F.; Krska, R.; Sulyok, M.

Sugar cane, *Saccharum officinarum*, is a tropical tall perennial grass used to produce raw sugar, molasses (brownish black viscous syrup), and ethanol in addition to the grass left over (bagasse) which is used as animal feed. In Africa, sugar cane is the second most cultivated crop after cassava. In Egypt, around 97% of the total sugar cane production, 15.8 million tons in 2013, is cultivated in the upper part of the country. Sugar cane juice is considered the most popular fresh juice in Egypt where cane juice shops are spreading through all the Egyptian cities. Few previous studies from African and Asian countries discussed the isolation of different fungal species from the plant. No reports have been published for the natural mycotoxin occurrence in cane grass and juice. Moreover, no regulations in Egypt or other countries for this commodity have been set.

Therefore, the aim of the present study was to screen for toxic fungal and bacterial metabolites in sugar cane grass (*n* = 21) and juice (*n* = 40) sold in Assiut city, Egypt. Quantification of the target analytes has been done using liquid chromatography-tandem mass spectrometry (LC-MS/MS).

Overall, 33 different metabolites in juice and 29 in cane grass have been quantified. The contamination with major mycotoxins such as aflatoxin B1 and aflatoxin G1 in juice was 23 (58%) and 7 (18%) and in grass was 10 (48%) and 2 (10%), respectively. The other most prevalent metabolites in juice were asperphenamate, tryptophol, emodin, citreorosein, ilicicolin B, versicolorin C, averufin, and iso-rhodoptilometrin while in cane grass, asperphenamate, emodin, tryptophol, citreorosein, iso-rhodoptilometrin, n-benzoyl-phenylalanine and kojic acid were the commonly detected ones. For exposure assessment, online and paper based questionnaire forms are being distributed to be filled in by Assiut city inhabitants (current sample size *n* = 66). The participants were of different ages and levels of education of both sexes. The preliminary results showed that more sugar cane juice consumption and consequently mycotoxins exposure occur in summer season and male inhabitants consume more than female.

In conclusion, follow-up detection studies in summer and in other cities of the country will provide better insights regarding the number of contaminating mycotoxins and their quantities in both sugar cane grass and juice.

### 3.45. Detoxification Efficacy of FreeTox towards DON and Its Masked Metabolites in Piglets

Goderis, A.; Van de Mierop, K.; Jin, L.; Michiels, J.

Mycotoxins are toxic fungal metabolites that can contaminate a wide array of cereals. One of the most prevalent mycotoxins is the trichothecene deoxynivalenol (DON). The toxic effects induced by DON are well characterized in all animal species, with pigs being the most susceptible. Occasionally, symptoms due to intake of mycotoxin-contaminated feed are more serious than what would be expected based on the feed contamination level. This observation has led to the discovery of masked mycotoxins which escape detection by routine analytical methods. Consequently, this would imply an underestimation of the degree of contamination upon analysis. Masked metabolites of DON include 3-acetyl-DON (3ADON), 15-acetyl DON (15ADON) and deoxynivalenol-3-β-d-glucoside (DON3G). These may have a direct toxic effect, or may be hydrolyzed to DON in the digestive tract of animals, resulting in higher exposure levels to DON. Another point of concern is the potential synergistic effect when DON and its metabolites are simultaneously present. An *in vivo* trial was set up to investigate the detoxification capacity of the mycotoxin binder FreeTox when pigs were fed a diet contaminated with a mixture of DON, 3ADON and 15ADON. It was hypothesized that FreeTox, which contains specific clay minerals and yeast derived cell walls, could reduce DON-induced growth depression. A total of 120 piglets, weaned at 3.5 weeks old, were assigned to 20 pens. From day 1 until 14 of the experiment, the diet was contaminated with 3 ppm of a mixture of DON (2.6 ppm), 3ADON (0.1 ppm) and 15ADON (0.3 ppm), after which the contamination level was reduced to 1 ppm from day 15 until 37. The piglets were assigned to one out of four dietary treatments: (T1) control uncontaminated diet, (T2) uncontaminated diet + 1 g FreeTox per kg feed, (T3) diet contaminated with DON mixture and (T4) diet contaminated with DON mixture + 1 g FreeTox per kg feed. During the first 14 days of the experiment, the pigs that received the DON-contaminated feed without FreeTox (T3) significantly consumed less feed when compared to pigs that received the same DON-contaminated feed with FreeTox (T4) (227 *vs*. 272 g/d; *p* = 0.039) which resulted in significant growth depression (159 *vs*. 205 g/d; *p* = 0.024). From day 15 until 37 and over the total experimental period, no significant differences were seen between the different treatments. However, numerically the same trend was seen when compared to the first 14 days. In conclusion, FreeTox alleviated the negative effects induced by DON and DON-metabolites in piglets.

### 3.46. Silanized Liposomes Loaded with Quantum Dots as Label for the Detection of Mycotoxins

Goryacheva, O.A.; Sobolev, A.M.; Foubert, A.; De Saeger, S.; Goryacheva, I.Y.; Beloglazova, N.V.

Nowadays different labels are developed for application in immunochemical assays in order to reach the highest sensitivity. Quantum dots (QDs) are one of the most promising labels because of their unique spectral properties. Their stable photoluminescence makes QDs ideal nanoprobes for chemical, biomedical and therapeutic labeling and imaging. However, when QDs are synthesized they are hydrophobic, only soluble in organic solutions and as such not applicable as label in assays. To solve this QDs can be loaded into liposomes.

The spherical double-layer structure of liposomes allows encapsulation of both hydrophobic and hydrophilic compounds and makes them biocompatible. Until now, a major drawback is the lack of stability in experimental conditions. This could result in leakage of compounds out of the liposomes.

In this research liposomes, loaded with QDs, were covered with a silica coating in order to increase the stability of the liposomes during storage and application and to decrease non-specific interaction. The silica coating also made bioconjugation with antibodies possible. Here, the hydrophilized QDs were coupled with antibodies against two mycotoxins (aflatoxin B1 (AFB1) and zearalenone (ZEN)). This allowed the performance of simultaneous detection of AFB1 and ZEN in cereals by FLISA. The silanized liposomes loaded with QDs allowed a significant increase of label stability, decrease of non-specific interaction and an increase of the assay sensitivity.

### 3.47. A Decision Support System to Control Mycotoxin Contamination in Maize Silages

Vandicke, J.; Debevere, S.; Fievez, V.; Croubels, S.; De Saeger, S.; Audenaert, K.; Haesaert, G.

Mycotoxins are toxic secondary metabolites produced by a variety of fungal species, such as *Fusarium*, *Penicillium* or *Aspergillus*, among others. Contamination of feed with mycotoxins can cause severe health problems in dairy cattle. Especially high yielding dairy cows with a high feed uptake and rapid ruminal flow are susceptible to gastroenteritis, reduced reproduction and reduced milk production, as a result of mycotoxin contamination. Maize silage is one of the main components of dairy feed in the region of Flanders, Belgium, and is therefore one of the main sources for mycotoxin uptake in dairy cows. This research aims towards providing dairy farmers in Flanders with a user-friendly prediction model, able to foresee mycotoxin contamination based on weather, cultivation, harvest and silage conditions. This model will be constructed based on analyses of maize silages across Flanders, and on our research focusing on methods to prevent mycotoxin contamination. One hundred maize silages will be selected based upon geographical spread, cultivation technique and silage conditions. These silages will be sampled once during harvest and 2–3 times during feeding every year for four years, and analyzed for mycotoxin and fungal contamination. Our research will be divided into five separate work packages, with the following topics: biofumigation of the soil using green crop manures, treatment of crop residues with antagonistic microbial populations, impact of harvest date and dry matter content on mycotoxin contamination, microbial detoxification in the silage, and toxicity of mycotoxins in dairy cattle. These results will aid in constructing and validating the prediction model.

Acknowledgements: this project is co-financed by Flanders Innovation & Entrepreneurship (VLAIO).

### 3.48. Identification and Characterization of Alternaria Species Causing Early Blight on Potato in Belgium

Vandecasteele, M.; Landschoot, S.; Audenaert, K.; Höfte, M.; De Saeger, S.; Haesaert, G.

*Alternaria* species, including *A. solani* and *A. alternata* are a serious threat for potato cultivation since heavy infections can lead to significant yield and quality losses. Both species cause necrotic symptoms, which cannot be visually distinguished. Over the past years, both pathogens have become increasingly important in NW Europe. Although the exact cause for this emerging problem remains elusive, it might be attributed to the combined effect of climate change, a reduced use of the fungicide mancozeb, the increased specificity of active ingredients to control *Phytophtora infestans* and the production of high-yielding susceptible cultivars. Furthermore, little is known about the Belgian *Alternaria* population and the contribution of both *A. solani* and *A. alternata* to the disease. The main goal of this research is to identify the primary causal agent of potato Early Blight, to determine inter- and intraspecific diversity within the *Alternaria* population in Flanders, and to unravel the complex interaction between stress-related hormones and the *Alternaria* infection.

To achieve these objectives, 124 fields were monitored throughout Flanders from 2013 to 2015. Results of this disease survey unequivocally show that the disease incidence and—pressure for all seasons was low and no significant differences between regions could be found. In a second part, we identified the population structure at the species level on different time points during the growing season. Therefore, leaf samples were collected during all three growing seasons and using a microscopic and molecular approach, we concluded that *A. alternata*, rather than *A. solani*, was the predominant species at the beginning of the growing season, while *A. solani* became apparent at later time points. Additionally, the genetic diversity of a subset of small-spore isolates was explored using a multilocus sequence typing analysis based on three conserved genetic regions. Our results show a high degree of diversity among the population and hints at a complex of different species, including *A. tenuissima* and *A. arborescens*. The same subset of isolates is now being tested for their pathogenicity and toxin fingerprint using high-throughput *in vitro* infection assays and an LC-MS/MS method respectively. Based on the outcome of these analyses, a subset of isolates will be used to investigate the complex interaction between the disease progress and stress-related hormones such as ethylene or auxins. Indeed, previous research shows that ethylene is an important factor in *Alternaria* spore germination and that it is a key component in upstream signaling of Programmed Cell Death induced by host-specific A. *alternata f. sp. lycopersici* (AAL)-toxin.

### 3.49. Evaluation of Next Generation Liquid Chromatography—Single Quadrupole Mass Spectrometry for Screening and Quantitative Analysis of Multiple Mycotoxins in Foods

Van Hulle, M.; Claereboudt, J.; Lattanzio, V.M.T.; Ciasca, B.; Stead, S.; McCall, E.; Powers, S.; Visconti, A.

Mycotoxin detection is of great importance in regulated environments such as food and animal feed analysis. Maximum permitted levels for the major mycotoxins, namely aflatoxins (AFB_1_, AFB_2_, AFG_1_, AFG_2_), ochratoxin A (OTA), fumonisins (FB_1_, FB_2_), deoxynivalenol (DON), and zearalenone (ZEA) are set by the European legislation (1881/2006/EC, 1126/2007/EC). Indicative maximum levels for the sum of T-2 and HT-2 toxins have been recently issued in the Recommendation 2013/165/EU. Although not regulated, attention is paid to the occurrence of nivalenol (NIV), another *Fusarium* toxin that frequently occurs in cereals also in combination with DON. Analytical methods for determination of major mycotoxins in food matrices need to be sensitive, selective and robust to provide accurate data when applied for monitoring, risk assessment, quality control and research. Most of the reference methods currently used for quality control purposes are based on immunoaffinity columns (IAC). However, due to the specificity of the antibodies used in these columns towards individual mycotoxins, different methods have been developed for a single mycotoxin or for closely related mycotoxins. In the last decade, the commercial availability of multi-mycotoxin IACs has opened new frontiers to the analysis of these contaminants in food control laboratories.

A liquid chromatography/mass spectrometry (LC-MS) method was developed for the simultaneous determination of aflatoxins (B_1_, B_2_, G_1_, G_2_), OTA, fumonisins (B_1_ and B_2_), NIV, DON, ZEA, T-2 and HT-2 in cereal based foods. A double extraction approach, based on high speed blending with water followed by methanol was applied for the effective co-extraction of the 12 mycotoxins under investigation in 4 min. Multi-toxin immunoaffinity columns (Myco6in1+^TM^, Vicam) were used for cleanup of the extract. The simultaneous detection and quantification of the 12 mycotoxins was performed by LC-MS evaluating performances of a new mass detector based on single quadrupole technology (Acquity QDa Detector, Waters). Furthermore, mycotoxin fragmentation patterns obtained by collision induced dissociation (CID) were investigated to identify two characteristic masses per each mycotoxin. The resulting detection approach enabled the acquisition of quantitative and confirmatory information in a unique chromatographic run. Reliability of this approach was evaluated by establishing the repeatability of ion ratios (quantifier/qualifier ion) by repeated measurements in standard solution and matrix extracts. Method performances such as linearity range, recoveries from spiked samples, quantification limits were evaluated and proved the method to be suitable to assess with a single analysis, compliance of the selected food commodities with the EU maximum permitted or recommended levels for all regulated mycotoxins. The developed LC-MS detection approach could represent, in routine mycotoxin analysis, a cost-effective alternative tool to more sophisticated LC-MS/MS equipments.

### 3.50. Using Ion Mobility Mass Spectrometry and Collision Cross Section Areas to Elucidate the α and β Epimeric Forms of Glycosylated T-2 Toxin

Claereboudt, J.; Stead, S.; McCullagh, M.; Busman, M.; McCormick, S.; Crich, D.; Kato, T.; Lattanzio, V.; Maragos, C.

Toxigenic fungi often grow on edible plants, thus contaminating food and feed with fungal metabolites. Plants can alter the chemical structure of mycotoxins as part of their defence against xenobiotics. The extractable conjugated or non-extractable bound mycotoxins formed remain present in the plant tissue but are currently neither routinely screened for in food nor regulated by legislation, thus they may be considered “masked”. *Fusarium* species mycotoxins are prone to metabolism or binding by plants. Toxicological data are scarce, but several studies highlight the potential threat to consumer safety from these substances. In particular, the possible hydrolysis of masked mycotoxins back to their toxic parents during mammalian digestion raises concerns. Masked mycotoxins may also elude conventional analysis because of modified physicochemical properties. All of these effects may lead to a potential underestimation or overestimation of the total mycotoxin content of the sample.

In this study we report the use of High Definition Mass Spectrometry (HDMS) as a powerful tool for the separation and characterisation of α and β epimeric forms of glycoslated T-2 and related toxins. The α-glycosylated T-2 standard was isolated from *Blastobotrys muscicola* cultures following exposure to T-2 and the β form produced *via* chemical synthesis. HDMS is a combination of high resolution mass spectrometry and high efficiency ion mobility separation. Ion mobility spectrometry (IMS) is a rapid orthogonal gas separation phase technique which allows another dimension of separation to be obtained within an UPLC timeframe. Compounds can be differentiated based on their size, shape and charge. In addition, both precursor ion and fragment ion information can be simultaneously acquired in a single injection in an HDMS experiment, referred to as HDMS^E^. HDMS^E^ data not only provides additional peak capacity but also insights into the molecular characteristics of the analytes for example, the elucidation of different isomeric species and intra-molecular sites of protonation.

The ion mobility drift time data acquired under HDMS^e^ mode was used to calculate the collision cross section area (CSS) values for both precursor ion and fragments within the data processing software of 244.85 and 251.33 Angstroms for the α and β T-2 glycosides, respectively. The combination of CCS, retention time, exact mass and fragmentation information provides a unique characteristic signature for the compounds. The individual CCS values derived for the α and β epimers can be used to determine which epimeric form of the toxin is present in the sample and can serve as a valuable tool during toxicological and profiling studies.

## 4. Author Affiliations

- Abdallah, M.F., Assiut University, Assiut, Egypt
- Arndt, B., Westfälische Wilhelms-Universität Münster, Münster, Germany
- Arroyo-Manzanares, N., Ghent University, Ghent, Belgium
- Atanda, O., McPherson University Ogun State, Nigeria
- Atoui, A., Lebanese Atomic Energy Commission, Beirut, Lebanon
- Audenaert, K., Ghent University, Ghent, Belgium
- Baranyi, N., University of Szeged, Szeged, Hungary
- Baydar, T., Hacettepe University, Ankara, Turkey
- Becker, S., Westfälische Wilhelms-Universität Münster, Münster, Germany
- Beloglazova, N., Ghent University, Ghent, Belgium
- Bencsik, O., University of Szeged, Szeged, Hungary
- Bhatnagar, D., USDA, New Orleans, LA, USA
- Bonin, L., Arvalis, Boigneville, France
- Busman, M., USDA-ARS National Center for Agricultural Utilization Research Peoria, Illinois, USA
- Callebaut, A., CODA-CERVA (Veterinary and Agrochemical Research Center), Tervuren, Belgium
- Calvo, A., Northern Illinois University, Dekalb, IL, USA
- Camenzuli, L., RIKILT, Wageningen University and Research centre, Wageningen, the Netherlands
- Cano-Sancho, G., University of California at Davis, Davis, USA
- Caron LF, Universidade Federal do Parana, Curitiba, Brazil
- Decleer, M., Ghent University, Ghent, Belgium
- Cary, J.W., USDA, New Orleans, LA, USA
- Chilaka, C.A., Ghent University, Ghent, Belgium
- Ciasca, B., National Research Council of Italy, Bari, Italy
- Claereboudt, J., Waters NV/SA, Zellik, Belgium
- Claes, J., Faculty of Engineering Technology, KU Leuven, Geel, Belgium
- Cramer, B., Westfälische Wilhelms-Universität Münster, Münster, Germany
- Crich, D., Wayne State University, Detroit, USA
- Croubels, S., Ghent University, Merelbeke, Belgium
- Cujic, S., SP Laboratorija AD, Becej, Serbia
- Cverheec, C., Université de Toulouse, INP-ENSAT, Castanet-Tolosan, France
- Dauthieux, F., Arvalis, Thiveral-Grignon, France
- De Baere, S., Ghent University, Merelbeke, Belgium
- De Boevre, M., Ghent University, Ghent, Belgium
- De Gelder, L., Ghent University, Ghent, Belgium
- De Middeleer, G., Ghent University, Ghent, Belgium
- De Ruyck, K., Ghent University, Ghent, Belgium
- De Saeger, S., Ghent University, Ghent, Belgium
- De Zutter, N., Ghent University, Ghent, Belgium
- Debevere, S., Ghent University, Merelbeke, Belgium
- Devreese, M., Ghent University, Merelbeke, Belgium
- Dhaouadi, S., Impextraco NV, Heist-op-den-Berg, Belgium
- Diana Di Mavungu, J., Ghent University, Ghent, Belgium
- Dubruel, P., Ghent University, Ghent, Belgium
- Ducerf, R., Arvalis, Boigneville, France
- Dzhavakhiya, V.G., All-Russian Research Institute of Phytopathology, Bolshie Vyazemy, Russia
- Ehrlich, K.C., Tulane University, New Orleans, LA, USA
- El Khoury, A., Université Saint-Joseph, Mkalles-Liban
- El Khoury, R., Université Saint-Joseph, Mkalles-Liban
- Elliott, C.T., Queen’s University Belfast, Belfast, United Kingdom of Great Britain and Northern Ireland
- Etoa, F.-X., University of Yaoundé I, Cameroon
- Farkas, H., SP Laboratorija AD, Becej, Serbia
- Fievez, V., Ghent University, Ghent, Belgium
- Fokou, E., University of Yaoundé I, Cameroon
- Fotso, M., Centre for Food and Nutrition Research, Yaoundé, Cameroon
- Foubert, A., Ghent University, Ghent, Belgium
- Girgin, G., Hacettepe University, Ankara, Turkey
- Goderis, A., AGRIMEX nv, Lille, Belgium
- Goftman, V., Saratov State University, Saratov, Russia
- Gong, Y.Y., Queen’s University Belfast, Belfast, United Kingdom of Great Britain and Northern Ireland
- Goryacheva, I., Saratov State University, Saratov, Russia
- Goryacheva, O.A., Saratov State University, Saratov, Russia
- Haesaert, G., Ghent University, Ghent, Belgium
- Heyndrickx, E., Ghent University, Ghent, Belgium
- Heyndrickx, E., Ghent University, Ghent, Belgium
- Hickert, S., Westfälische Wilhelms-Universität Münster, Münster, Germany
- Hofgaard, I.S., Norwegian Institute of Bioeconomy Research, Ås, Norway
- Höfte, M., Ghent University, Ghent, Belgium
- Hoogendoorn, K., Plant Research International, Wageningen University and Research centre, Wageningen, the Netherlands
- Humpf, H.-U., Westfälische Wilhelms-Universität Münster, Münster, Germany
- Huybrechts, B., CODA-CERVA (Veterinary and Agrochemical Research Center), Tervuren, Belgium
- Huybrechts, I., International Agency for Research on Cancer, Lyon, France
- Jaksic, M., SP Laboratorija AD, Becej, Serbia
- Janson, J.P., FNAMS, Troyes, France
- Jin, L., Ghent University, Ghent, Belgium
- Kalinina, S., Westfälische Wilhelms-Universität Münster, Münster, Germany
- Karaoglanidis, G.S., Aristotle University of Thessaloniki, Thessaloniki, Greece
- Kato, T., Wayne State University, Detroit, USA
- Kiebooms, J.A.L., CODA-CERVA (Veterinary and Agrochemical Research Center), Tervuren, Belgium
- Kilicarslan, B., Hacettepe University, Ankara, Turkey
- Kocsubé, S., University of Szeged, Szeged, Hungary
- Köhler, J., Westfälische Wilhelms-Universität Münster, Münster, Germany
- Koppenol, A., Impextraco NV, Heist-op-den-Berg, Belgium
- Kovalsky, P., BIOMIN Holding GmbH, Herzogenburg, Austria
- Kowalski, E., Ghent University, Merelbeke, Belgium
- Krska, R., University of Natural Resources and Life Sciences, Vienna, Austria
- Krug, I., Westfälische Wilhelms-Universität Münster, Münster, Germany
- Labreuche, J., Arvalis, Boigneville, France
- Landschoot, S., Ghent University, Ghent, Belgium
- Lattanzio, V.M.T., National Research Council of Italy, Bari, Italy
- Laurain, J., OLMIX, Bréhan, France
- Lauwers, M., Ghent University, Merelbeke, Belgium
- Leclere, A., Arvalis, Boigneville, France
- Letor, B., Innovad, Antwerpen-Berchem, Belgium
- Logrieco, A.F., Research National Council, Bari, Italy
- Machado, P., Junior, Impextraco Latin America, Curitiba, Brazil
- Madder, A., Ghent University, Ghent, Belgium
- Maragos, C., USDA-ARS National Center for Agricultural Utilization Research Peoria, Illinois, USA
- Marengue, E., LABOCEA public lab, Ploufragan, France
- Marín, S., University of Lleida, Lleida, Spain
- Marinkovic, D., SP Laboratorija AD, Becej, Serbia
- Marosanovic, B., SP Laboratorija AD, Becej, Serbia
- Maroun, R., Université Saint-Joseph, Mkalles-Liban
- Mathieu, F., Université de Toulouse, INP-ENSAT, Castanet-Tolosan, France
- Maumené, C., Arvalis, Boigneville, France
- Maunas, L., Arvalis, Montardon, France
- McCall, E., Waters Corporation, Manchester, UK
- McCormick, S., USDA-ARS National Center for Agricultural Utilization Research Peoria, Illinois, USA
- McCullagh, M., Waters Corporation, Wilmslow, UK
- Medema, M., Plant Research International, Wageningen University and Research centre, Wageningen, the Netherlands
- Medoua, G.N., Centre for Food and Nutrition Research, Yaoundé, Cameroon
- Meleard, B., Arvalis, Boigneville, France
- Mengelers, M., RIVM, Bilthoven, the Netherlands
- Michiels, J., Ghent University, Ghent, Belgium
- Miles, C.O., Norwegian Veterinary Institute, Oslo, Norway
- Molassiotis, A., Aristotle University of Thessaloniki, Thessaloniki, Greece
- Mooney, M.H., Queen’s University Belfast, Belfast, United Kingdom of Great Britain and Northern Ireland
- Muccio, M., BIOMIN Holding GmbH, Herzogenburg, Austria
- Myresiotis, C., Aristotle University of Thessaloniki, Thessaloniki, Greece
- Nacer Khodja, E., OLMIX, Bréhan, France
- Naehrer, K., BIOMIN Holding GmbH, Herzogenburg, Austria
- Ngoko, Z., Catholic University of Cameroon, Bamenda, Cameroon
- Nguegwouo, E., University of Yaoundé I, Cameroon
- Njobeh, P.B., University of Johannesburg, Johannesburg, South Africa
- Njumbe Ediage, E., Ghent University, Ghent, Belgium
- Oplatowska-Stachowiak, M., Queen’s University Belfast, Belfast, United Kingdom of Great Britain and Northern Ireland
- Orlando, B., Arvalis, Boigneville, France
- Osteresch B, Westfälische Wilhelms-Universität Münster, Münster, Germany
- Powers S, VICAM, Milford, MA, USA
- Rajkovic A, Ghent University, Ghent, Belgium
- Ramos, A.J., University of Lleida, Lleida, Spain
- Rise, F., University of Oslo, Oslo, Norway
- Robin, N., Arvalis, Montardon, France
- Sajic, N., EuroProxima B.V., Arnhem, Netherlands
- Sanchis, V., University of Lleida, Lleida, Spain
- Sas, B., Ghent University, Ghent, Belgium
- Schelfhout, K., OVAM, Mechelen, Belgium
- Schelstraete, W., Ghent University, Merelbeke, Belgium
- Schmidt, H.S., Westfälische Wilhelms-Universität Münster, Münster, Germany
- Shcherbakova, L.A., All-Russian Research Institute of Phytopathology, Bolshie Vyazemy, Russia
- Sioen, I., Ghent University, Ghent, Belgium
- Smagghe, G., Ghent University, Ghent, Belgium
- Sobolev, A.M., Saratov State University, Saratov, Russia
- Vandicke, J., Ghent University, Ghent, Belgium
- Stanic ,A., Norwegian Veterinary Institute, Oslo, Norway
- Statsyuk, N.V., All-Russian Research Institute of Phytopathology, Bolshie Vyazemy, Russia
- Stead, S., Waters Corporation, Manchester, UK
- Stroka, J., IRMM Geel, Geel, Belgium
- Studt, L., University of Natural Resources and Life Sciences, Vienna, Austria
- Sulyok, M., University of Natural Resources and Life Sciences, Vienna, Austria
- Suman, M., Barilla Advanced Laboratory Research, Parma, Italy
- Szekeres, A., University of Szeged, Szeged, Hungary
- Tangni, E.K., CODA-CERVA (Veterinary and Agrochemical Research Center), Tervuren, Belgium
- Tanou, G., Aristotle University of Thessaloniki, Thessaloniki, Greece
- Testempasis, S., Aristotle University of Thessaloniki, Thessaloniki, Greece
- Thiry, C., CODA-CERVA (Veterinary and Agrochemical Research Center), Tervuren, Belgium
- Tudzynski, B., Westfälische Wilhelms-Universität Münster, Münster, Germany
- Udovicki, B., University of Belgrade, Belgrade, Serbia
- Uhlig, S., Norwegian Veterinary Institute, Oslo, Norway
- Uka, V., Ghent University, Ghent, Belgium
- Vágvölgyi, C., University of Szeged, Szeged, Hungary
- Valade, R., Arvalis, Thiveral-Grignon, France
- Van Bocxlaer, J., Ghent University, Ghent, Belgium
- Van de Mierop, K., AGRIMEX nv, Lille, Belgium
- Van der Fels-Klerx, H.J., RIKILT, Wageningen University and Research centre, Wageningen, the Netherlands
- Van der Lee, T.A.J., Plant Research International, Wageningen University and Research centre, Wageningen, the Netherlands
- Van Hulle, M., Waters NV/SA, Zellik, Belgium
- Vandecasteele, M., Ghent University, Ghent, Belgium
- Vanhaecke, L., Ghent University, Merelbeke, Belgium
- Vanhoutte, I., Ghent University, Ghent, Belgium
- Varga, J., University of Szeged, Szeged, Hungary
- Verheijen, R., EuroProxima B.V., Arnhem, Netherlands
- Vidal, A., University of Lleida, Lleida, Spain
- Visconti, A., National Research Council of Italy, Bari, Italy
- Vitry, C., Arvalis, Thiveral-Grignon, France
- Voinova, T.M., All-Russian Research Institute of Phytopathology, Bolshie Vyazemy, Russia
- Waalwijk, C., Plant Research International, Wageningen University and Research Centre, Wageningen, The Netherlands
- Wauters, J., Ghent University, Merelbeke, Belgium
- Wiemann, P., University of Wisconsin-Madison, Madison, WI, USA
- Willems, B., Innolab CVBA, Ghent, Belgium
- Xu, Y., Queen’s University Belfast, Belfast, United Kingdom of Great Britain and Northern Ireland
- Zheng, H., Ghent University, Ghent, Belgium

## Figures and Tables

**Table 1:** Number and distribution (%) of sclerotia in soil profile after one tillage (L: plow + rotative harrow or WS: Simplified tillage, Lemken Smaragd 9 + harrow) or two tillages in sequence L/L, L/WS, or WS/L. The depth of tillage with the cultivator Lemken Smaragd 9 is set to 10 cm, and 20 cm with the plough. The sclerotia were not searched for below 10 cm after simplified tillage.

Number of sclerotia/m^2^ and distribution in (%) per layer		0–5 cm	5–10 cm	10–15 cm	15–20 cm	Total
	*After tillage: July 2013*					
July 2013	L	15.3 (7%)	19.0 (9%)	75.3 (36%)	100.3 (48%)	210.0 (100%)
	WS	103.3 (65%)	56.3 (35%)	0.0 (0%)	0.0 (0%)	159.7 (100%)
	*After tillage: October 2014*					
October 2014	L/L	17.3 (30%)	16.7 (29%)	10.7 (19%)	12.3 (22%)	57.0 (100%)
	L/WS	0.7 (1%)	8.7 (18%)	18.0 (36%)	22.0 (45%)	49.3 (100%)
	WS/L	1.3 (7%)	5.7 (29%)	5.7 (29%)	7.0 (35%)	19.7 (100%)

